# Food Anticipatory Activity Behavior of Mice across a Wide Range of Circadian and Non-Circadian Intervals

**DOI:** 10.1371/journal.pone.0037992

**Published:** 2012-05-25

**Authors:** Matthew D. Luby, Cynthia T. Hsu, Scott A. Shuster, Christian M. Gallardo, Ralph E. Mistlberger, Oliver D. King, Andrew D. Steele

**Affiliations:** 1 Division of Biology, California Institute of Technology, Pasadena, California, United States of America; 2 Department of Psychology, Simon Fraser University, Burnaby, British Columbia, Canada; 3 Boston Biomedical Research Institute, Watertown, Massachusetts, United States of America; Pennsylvania State University, United States of America

## Abstract

When rodents are fed in a limited amount during the daytime, they rapidly redistribute some of their nocturnal activity to the time preceding the delivery of food. In rats, anticipation of a daily meal has been interpreted as a circadian rhythm controlled by a food-entrained oscillator (FEO) with circadian limits to entrainment. Lesion experiments place this FEO outside of the light-entrainable circadian pacemaker in the suprachiasmatic nucleus. Mice also anticipate a fixed daily meal, but circadian limits to entrainment and anticipation of more than 2 daily meals, have not been assessed. We used a video-based behavior recognition system to quantify food anticipatory activity in mice receiving 2, 3, or 6 daily meals at intervals of 12, 8, or 4-hours (h). Individual mice were able to anticipate as many as 4 of 6 daily meals, and anticipation persisted during meal omission tests. On the 6 meal schedule, pre-prandial activity and body temperature were poorly correlated, suggesting independent regulation. Mice showed a limited ability to anticipate an 18 h feeding schedule. Finally, mice showed concurrent circadian and sub-hourly anticipation when provided with 6 small meals, at 30 minute intervals, at a fixed time of day. These results indicate that mice can anticipate feeding opportunities at a fixed time of day across a wide range of intervals not previously associated with anticipatory behavior in studies of rats. The methods described here can be exploited to determine the extent to which timing of different intervals in mice relies on common or distinct neural and molecular mechanisms.

## Introduction

The ability of animals to time intervals of long and short duration has been studied in two major traditions, chronobiology and comparative cognition. Chronobiologists, dating to Richter (Richter, 1922), have been concerned with evidence for timing of events, such as scheduled meals, that are linked to time of day. In the canonical paradigm, a rat is fed once daily at a fixed time, typically in the middle of the light period, when activity levels in nocturnal rodents are normally low [1], [2], [3], [4]. Within a few days of restricted feeding, the rat becomes active an hour (h) or more prior to mealtime, and the intensity of activity (measured by running wheels, operant levers or food bin motion sensors) rises monotonically to a peak at mealtime. If food is withheld then the bout of food anticipatory activity (FAA) extends until after the usual end of the mealtime, and reappears at the appropriate time on subsequent days of food deprivation [5]. Rats can anticipate meals that occur at intervals as long as 28 h, and as short as 23 h, but anticipation has been reported to fail outside of that range [3], [6], [7], [8], [9]. Thus, anticipation of a daily meal has properties of a rhythm generated by a self-sustaining, entrainable oscillator or clock that has an endogenous periodicity of about 24 h (i.e., circadian). Notably, this rhythm is not affected by ablation of the master, light-entrainable circadian pacemaker in the suprachiasmatic nucleus (SCN), suggesting the existence of a separate food-entrainable circadian oscillator [3], [10].

The field of comparative cognition has a similarly long history, dating back to the early behaviorist formulations of learning theory to explain patterns of operant responding of rats on reinforcement schedules [11]. A canonical paradigm in this discipline is the fixed interval feeding schedule, in which food (or some other reward) is provided contingent on an operant behavior emitted after some fixed, arbitrary interval, typically in the seconds (s) to minutes (min) range, following a previous reward or an environmental stimulus. Anticipatory responding under these short interval schedules typically fails to persist beyond one cycle if the reward is withheld. Renewed responding may occur after some delay, but the original interval is not preserved, can be reset immediately if the conditioned stimulus or fixed interval reward is delivered early or late, and exhibits proportionality between the duration of the bout of anticipation, and the duration of the interval being timed (longer bouts of anticipation for longer intervals, scaling linearly) [12], [13]. These properties have inspired timing models by which rats measure elapsed time using, e.g., a neural pulse emitter and counter [14], [15].

The distinct properties of circadian and short-interval timing suggest that different mechanisms have evolved or have been adapted to solve different timing problems encountered in the natural world. For example, CLOCK mutant mice appear to have intact interval timing abilities [16]. Questions arise about time intervals in between those that span hours as opposed to full days or min. Such timing intervals have received comparatively less attention. Chronobiological work has yielded consistent evidence that rats can anticipate two daily meals at fixed times of day anywhere from 6–18 h apart, and may be able to track two daily meals with slightly different circadian periods (e.g., 24 h and 24.5 h) [17], [18], [19]. One study provided evidence that rats in running wheel cages fail to anticipate more than 2 of 3 daily mealtimes 6–12 h apart on a given day [18]. These results imply that the food-entrainable circadian oscillators can be dissociated into two independently entrainable cohorts capable of driving separate bouts of anticipatory activity at circadian intervals, analogous to the two-oscillator organization of the SCN circadian pacemaker [18]. Rats provided two daily meals can be trained to look for food in one place at one mealtime and in another place at a second mealtime, using circadian phase as a time cue [20], [21], [22]. Birds can make at least 4 distinct time-place associations using endogenous time of day cues [23]. Finally, bees can anticipate 24 h but not 19 h feeding intervals and can anticipate more than one meal per day [2]. These observations suggest an alternative model, wherein meal anticipation and time-place learning are based on the ability to discriminate and remember multiple phases of a single clock. Additional cognitive processing would be required for animals to use one clock to time two meals with different circadian periodicities.

In the comparative cognition tradition, timing of intervals in the hourly range has received little attention. There is at least one report that rats can anticipate meals at long intervals (e.g., 14 h, 16 h, 20 h) that are not multiples of 24 h [24], [25], [26]. This provides evidence for non-circadian oscillators with endogenous periodicities in the 14 h and 20 h range. There is also one report that the duration of anticipation of a single nocturnal mealtime exhibits the scalar property; the anticipation waveforms for meals 3 or 7 h after lights-off superimposed when the time scales were normalized [24]. Whether timing was dependent on the lights-off signal was not assessed. Given the repeated demonstration that anticipation of a daily meal persists in constant dark or light, these results imply that anticipation of a daily meal can be jointly controlled by a circadian mechanism and by a non-circadian, environmental cue-based interval timing process that can operate at intervals of up to 7 h.

If there are non-circadian oscillators and interval timers capable of producing anticipatory behavior at intervals ranging from 7 to 20 h, then some explanation is needed for the observed failure of rats to anticipate feeding schedules in the 18 to 23 h range [3], [8], [17], [27], or to stably anticipate more than two of three daily meals at 6–12 h intervals [28]. A critical factor may be the behaviors that are measured as assays of meal anticipation. For example, anticipation of long but non-circadian intervals of less than a day has been reported in nose poking behavior [24], but not in wheel running [6], [9], [27].

**Figure 1:**
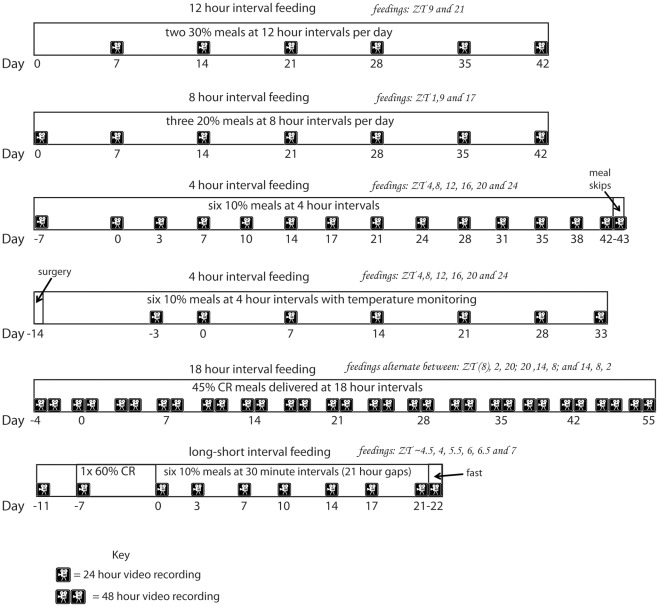
Diagram of experiments. Each experiment is represented by a rectangle proportional to the duration of the experiment. Video-recordings are indicated by a camera symbol and days on which recordings occurred are numbered. Feeding times are indicated in italics; for the 18 h interval feeding experiment there were three alternating sets of feeding times. For the first type, the first feeding event is when the video recording begins at ZT 8 and thus anticipation of this feeding event is not possible so we indicated that feeding event in parenthesis.

To move toward a resolution of these issues, we have utilized a video based, semi-automated behavior recognition system [29] to provide a richer behavioral assay to detect anticipatory behavior prior to 2, 3 or 6 daily meals presented at 12, 8 or 4 h intervals, respectively. A second 6-meal experiment was conducted to measure core body temperature to determine if the timing mechanism controlling behavioral anticipation was tightly coupled to metabolic processes. An 18-h feeding schedule was used to assess anticipation of meals recurring at non-circadian intervals and variable but non-random times of day. To establish a model for concurrent testing of intervals in the mins, hours and circadian range, we also examined anticipatory behavior when food was provided in 6 small meals, provided at 30 min intervals during a fixed, 2.5 h daily window (Figure 1 summarizes the entire experimental design). Finally, as a prerequisite to molecular genetic approaches to study the neuronal basis of FAA, we utilized inbred C57BL/6J mice rather than rats. Mice readily anticipate a single daily meal [10], and are capable of anticipating two daily meals in wheel running behavior [30], but there are no data available on feeding schedules providing 3 or more meals per day, or one meal at non-circadian intervals. We found that mice are capable of anticipating 2 or 3 meals per day, and that individual mice can anticipate as many as 4 of 6 meals on a given day. Persistence of anticipatory cycles following meal omission tests appears to rule out cue-based interval timing as a possible mechanism. We also observed concurrent circadian and short-interval anticipatory behavior in mice receiving 6 meals at 30 min intervals at a fixed time of day. These results establish the mouse as a model for behavioral timing of events at intervals ranging from 30 min to 24 h and suggest a mechanism other than cue-based interval timing.

## Results

### Two-meal calorie restricted feeding schedule

**Figure 2:**
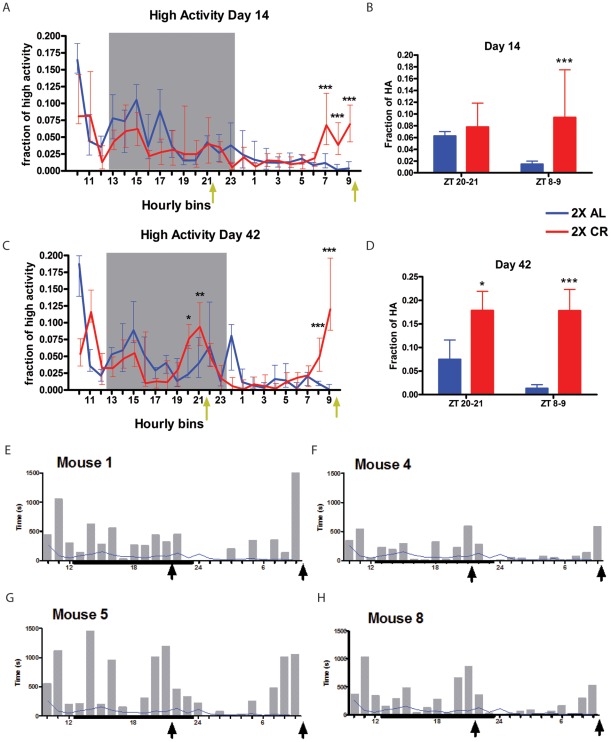
12 h interval feeding schedule. (A) Normalized median +/− IQR high activity data on day 14 of 2X feeding. The normalization is equivalent to dividing the number of frames during which high intensity activity (walking, hanging, jumping, or rearing) is observed per h by the number of frames the high intensity activity is observed during the duration of the 24 h video, yielding a fraction of high activity per hourly bin. Each ZT time represents the total number of s of high activity in the h preceding it (eg. The data point at ZT 9 comprises the amount of high activity that occurred from ZT 8:00-ZT 8:59. (B) The amount of normalized high activity in the 2 h preceding each feeding on day 14. (C) Normalized median +/− IQR high activity data on day 42 of 2X feeding. (D) The amount of normalized high activity in the 2 h preceding each feeding on day 42. Statistical significance was determined using the Mann-Whitney Test with asterisks denoting * = p<0.05, **  = p<0.01, *** = p<0.001. n = 8 for both 2X AL and 2X CR at all time points. Yellow arrows represent feeding times. Note: arrows are offset from feeding times to denote that the data point at the feeding time represents data from before the feeding event. For example, for a feeding at ZT21 the data point at ZT21 represents data from ZT20 to ZT21 and does not include time when food was present, as food was delivered at ZT21. Gray box indicates the 11h dark period. (E) Individual data from 2X CR mouse #1 on day 42, (F) for mouse #4, (G) mouse #5, and (H) mouse #8. Mean 2X AL high activity is sown as a blue line and gray bars indicate the amount of high activity occurring per one h bin for the CR mouse. Dark line indicates the 11 h dark period and arrows indicate feeding times.

We first sought to confirm that mice can anticipate two daily meals (Figure 1). Mice were fed 30% of their ad libitum (AL) food intake twice daily at ZT 9 and 21 for a total of 60% daily (13 h light: 11 h dark cycle; by convention ZT 12 is defined as “lights off”). Control mice with AL access to food received additional pellets with the same automated feeding devices twice per day to control for the disturbance caused by the automated feeder. Because 60% calorie restriction (CR) has the potential to change total activity, data were normalized by dividing the s of high activity (defined as hanging, jumping, rearing, and walking) in each hourly bin by the total amount per day, yielding a fraction of high activity occurring within each hourly bin. On day 14 of the 2X feeding experiment the CR cohort showed significant anticipation of the ZT9 but not the ZT21 meal (Figure 2A–B). Each data point represents the amount of high activity preceding that hourly bin; for example, the data at ZT 9 represents the amount of high activity from ZT 8 to 9 and the meal will be delivered just after this point. By day 42 of the 2X feeding regimen, there were two clear anticipatory activity peaks in the group data for both feedings, beginning at ZT 20 for the dark-cycle feeding and at ZT8 for the light-cycle feeding (Figure 2C–D). We quantified the amount of normalized high activity 2 h prior to each feeding event across the entire experiment and observed statistically significant anticipation of ZT9 feedings by day 7, and significant anticipation of the ZT21 feeding only on day 42 (Figure S1). Presumably this reflects either a difficulty in increasing activity deep into the dark cycle and/or a masking effect of the increased activity in AL control mice at this time.

Inspection of data from four exemplary individual mice (mouse #'s 1, 4, 5, and 8) on day 42 shows that activity was increased prior to both meals for several of the mice (Figure 2E–H). Many of the individual mice failed to predict one or both meals (the full dataset is presented in Figure S2). Because of the variability in activity levels between mice, and for individual mice in different time intervals, it is not always “clear-cut” whether a particular mouse anticipates a given meal. As a cutoff, we require a 50% increase in activity in a time interval preceding the feeding, leading to an activity level at least 25% higher than the population's median activity across the entire light- or dark-cycle, as appropriate. Details of this analysis method are given in the materials and methods section.

### Three-meal calorie restricted feeding schedule

**Figure 3:**
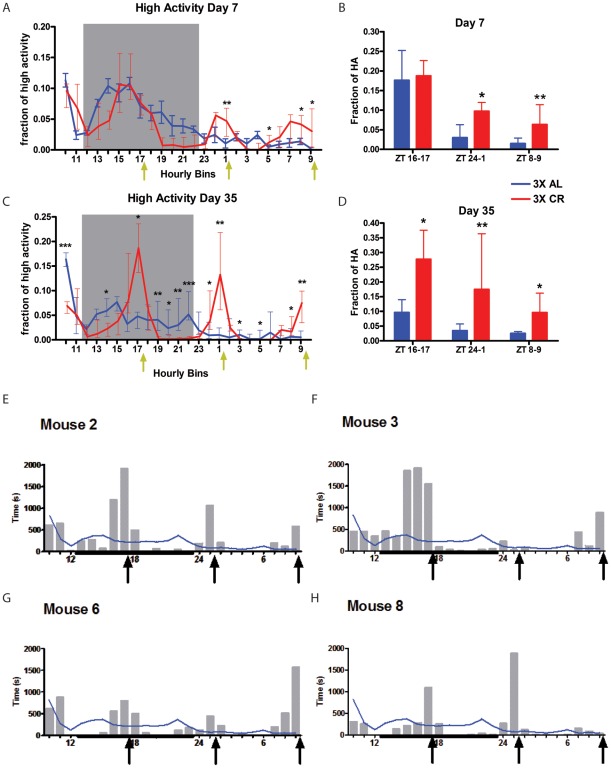
8 h interval feeding schedule. (A) Normalized median +/− IQR high activity data on day 7 of 3X feeding. (B) The amount of normalized high activity in the 2 h preceding each feeding event on day 7. (C) Normalized high activity data on day 35 of 3X feeding. (D) The amount of normalized high activity in the 2h preceding each feeding event on day 42. For 3X AL data n-6 on day 7 and n = 8 on day 35; for 3X CR, n = 8 on day 7 and 35. (E–H) Individual mouse data for 8 h interval feeding. The s of high intensity activity in each h for individual CR mice on days 35 3X CR feeding for (E) mouse #2, (F) mouse #3, (G) mouse #6, and (H) mouse #8. Black arrows represent feeding times. Weighted black line on the x-axis indicates the 11h dark cycle.

We next tested whether mice could anticipate three CR meals delivered at 8 h intervals. “3X CR” mice were fed 20% of their AL food intake (for a total of 60% daily) at ZT 17, 1, and 9 for 42 days, while “3X AL” control mice received food at the same times but already had several pellets in their food trough. Normalized high activity data after just seven days of 3X CR feeding revealed a small but significant increase in activity for both light cycle feedings (ZT 1 and 9) (Figure 3A–B). By the 35^th^ day of 3X CR, the median normalized high activity showed three striking peaks of activity preceding each feeding by about 2 h (Figure 3C–D). In the 3X CR group data, the fraction of normalized high intensity activity during the 2 h prior to the ZT 1 and ZT 9 mealtimes was significantly different from the AL control group as early as day 7 (Figure S3). The fraction preceding the ZT17 (nighttime) meal was not significant until day 28, but exceeded that evident at the other two mealtimes (Figure S3).

To determine the extent to which group data were representative of individual mice, we examined plots of the number of s of high activity for each mouse on day 35 of 3X CR feeding–the only time point for which all three meals showed significant FAA for group data. These plots revealed that most mice anticipated only two of the three meals times (Figure 3E–I; Figure S4). For example, mouse #3 showed a large increase in activity preceding the dark cycle feeding at ZT17, no anticipation for the following light cycle feeding at ZT1, and moderate anticipation for the second light cycle feeding at ZT9 (Figure 3F). Examining individual mouse data from different days on CR showed that some mice are better at predicting say, the feeding at ZT1 versus the feeding at ZT9 (Figure S4). On day 35 of CR, two of the mice, #2 and #6, had a moderate amount of FAA preceding each meal, suggesting that entrainment to feeding can occur in intervals as short at 8 h. On average, CR mice anticipated 1.5 of the 3 meals on day 7 (vs. 0.33 for AL mice; an increase with significance p = 0.004 by the Wilcoxon rank-sum test) and 2.5 of the 3 meals on day 35 (vs. 0.12 for AL mice; p  = 0.0002). In aggregate these results demonstrate that individual mice can anticipate at least 3 daily meals.

### Six-meal calorie restricted feeding schedule

**Figure 4:**
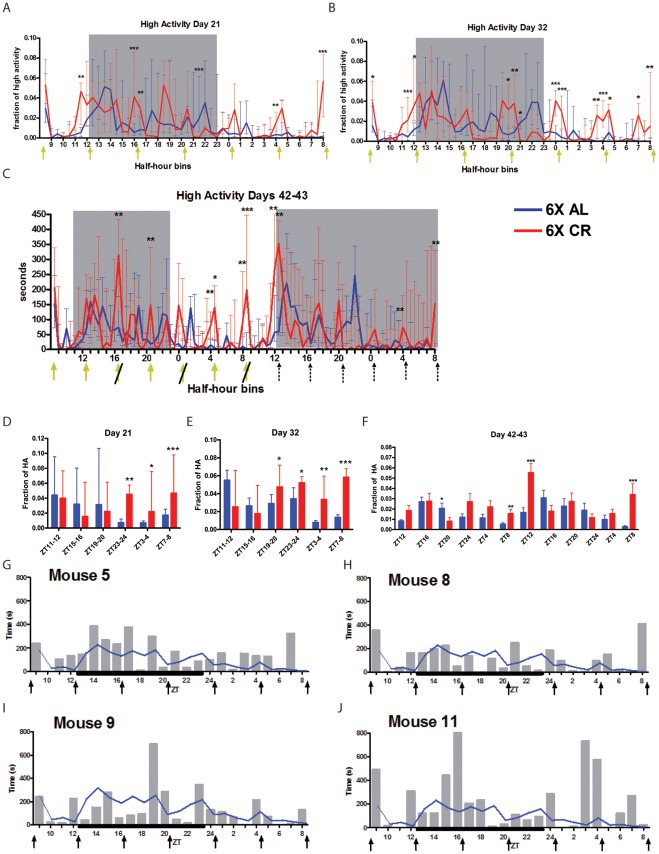
Four h interval feeding schedule. (A) Normalized median +/− IQR high activity data on day 21 of 6X feeding. (B) Normalized high activity data on day 32. (C) Normalized high activity data on days 42–43 CR feeding. (C) Normalized high activity data on days 42–43 of 6X feeding. (D–F) The fraction of high activity occurring 1h prior to each meal time for days (D) 21, (E) 32, and (F) 42–43 of 6X feeding. Yellow arrows with a slash through them indicate false feeding events where a small plastic pellet was delivered instead of food. Dashed arrows indicate 4h intervals and do not represent feeding (or false-feeding) events. n = 12–14 mice at for each group at each time point presented. (G–J) Individual mouse data for 4 h interval feeding. The s of high intensity activity in each h for (G) mouse # 5, (H) mouse #8, (I) mouse #9, and (J) mouse #11 on day 32 of 6X CR. Black arrows represent feeding times. Weighted black line on the x-axis indicates the 11 h dark cycle.

Given our evidence for anticipation of three daily meals in individual mice, we next sought to define an upper limit for temporal discrimination of daily mealtimes by providing mice with 6 daily meals. Each meal comprised 10% of AL food intake (for a total of 60% daily), at ZT 4, 8, 12, 16, 20, and 24 for 42 days. By day 21 of this feeding schedule, the 6X CR mice as a group showed six activity peaks that mostly preceded the feeding event (Figure 4A). Anticipation of the meals at ZT 24, 4, and 8 were most obvious as these meals occurred during the light period when 6X AL control activity was very low. Activity peaks prior to the three daytime meals were also salient on day 32 of 6X CR, but activity peaks prior to the three nighttime meals were less obvious (Figure 4B). On day 42 of the feeding schedule, three false feeding events were scheduled, during which a plastic pellet was delivered in lieu of a food pellet at ZT 16, 0, and 8 (Figure 4 C). To compensate for the missed meals and retain an overall 60% CR for day 42, the meals delivered preceding a false feeding equal to 20% of AL food intake (i.e. twice the size of typical 6x CR meals). The mice were then fasted for 24 h on day 43, in constant dark (DD) and without false feeding events. Activity built up in anticipation of false feedings and surprisingly returned to baseline levels quickly, before rising again several hours later in anticipation of the next feeding event (Figure 4C). During the 24 h fasting day in DD (day 43), small peaks of high activity were evident in the group data preceding most of the usual mealtimes. Although the peak level appeared to diminish, the timing of the peaks was remarkably consistent with the 4 h interval feeding schedule.

Analysis of normalized high activity during the 1 h preceding each feeding event on days 21, 32, and 42–43 confirms that feeding events at ZT 4, 8, and 24 were the most consistently anticipated, as activity among 6X CR mice was significantly higher than 6X AL controls at each of these time points (Figure 4D–F). Remarkably, missing two feeding events did not preclude proper timing of FAA for the ZT8 feeding of day 42, where we observed significant anticipation (Figure 4F). The next scheduled meal should have occurred at ZT12 and, again, the 6X CR mice significant FAA for this non-event but most of the following feedings in constant darkness were not anticipated significantly (Figure 4F). On average, CR mice anticipated 2.21 of the 6 meals on day 21 (vs. 1.31 for AL mice; p  = 0.02) and 3.6 of the 6 meals on day 32 (vs. 0.69 for AL mice; p  = 0.00002).

Overall, activity prior to the 3 nighttime meals was not consistently elevated compared to the AL control group (Figure S5). Although no single recording date revealed significant anticipation prior to every feeding time point in group data, each meal was significantly anticipated in at least one recording (Figure S5). Inspection of the data for each individual mouse revealed that individuals anticipated a variable number of meals, and that the meals anticipated also varied across days of recording (Figure S6).

### Body temperature and activity measurement during six-meal calorie restricted feeding schedule

To determine whether anticipation of multiple meals might be revealed more strongly in a continuous physiological variable, temperature sensitive transponders were implanted in the peritoneal cavity of 14 mice. The mice then received either the AL control diet (n = 7) or the 60% CR diet (n = 7) at ZT 4, 8, 12, 16, 20, and 24 for 33 days. Temperature measurements were made at 15 min intervals for the duration of the experiment and activity was measured by video recordings on a nearly weekly basis (Figure 1).

**Figure 5:**
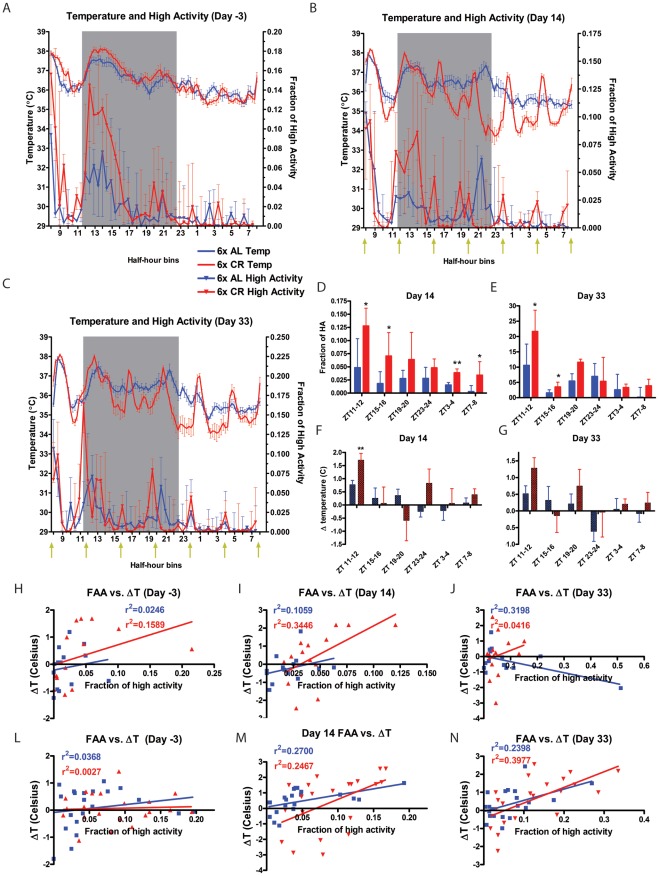
Body temperature and activity measurements during 4h interval feeding. (A) Mean body temperature (left y-axis and top of the panel) and normalized median high activity data (right y-axis and bottom of the panel panel) plotted against ZT time for day -3 (CR and AL groups are listed separately but no dietary manipulation has occurred at this point). (B) Temperature and activity after 14 days of 4 h interval feeding. (C) Temperature and activity after 33 days of 4 h interval feeding. Normalized high activity for 1h preceding each feeding event on day 14 (D) and day 33 (E). Change in temperature 1 h preceding each feeding event on day 14 (F) and Day 33 (G). Fraction of high activity plotted against change in temperature 1h preceding daytime (H–J) and nighttime (L–N) feeding events for days -3, 14 and 33. Least-square regression lines and r^2^ values are shown on the top left of each panel. Error bars correspond to SEM for temperature values, and to IQ ranges for activity values. Statistics were performed using t-test for temperature values and Mann-Whitney for behavior values. * denotes p<0.05, ** denotes p<0.01. n = 7 for AL and CR. n = 7 for both AL and CR at all time points. Yellow arrows indicate feeding times.

Three days prior to initiating the feeding schedule, the group average temperature waveforms were very similar although the mean level was elevated in the 6X CR group from ∼ZT12–16, when these mice were also more active compared to the 6X AL group (Figure 5A). Body temperature in both groups was increased at ZT 7.5–8, when the mice were handled after video recordings were terminated. By day 14 of the feeding schedule, the temperature and activity waveforms of mice on 6X CR had changed markedly (Figure 5B). Each mealtime was associated with a peak of activity preceding food delivery (statistically significant for meals ZT4, 8, 12, 16; Figure 5D), and a peak of temperature sometime within the h after meal onset. At the ZT 8, 12, 16 and 20 mealtimes, the temperature rise was initiated prior to mealtime, although this change was significant by comparison with the AL group only at the ZT12 mealtime. On day 33 of 6X feeding the body temperature rhythms of 6X AL control mice showed similarly marked peaks during mealtime, but the mealtime anticipatory component of these peaks was if anything less marked, particularly at the ZT20 and 24 feedings (Figure 5C).

Regression analysis revealed a surprisingly weak relationship between the amount of anticipatory activity and the change of body temperature during the h preceding the mealtimes. Data from the light period mealtimes (Figure 5H–J) and the dark period mealtimes (Figure 5L–N) were analyzed separately because daytime feeding events showed greater behavioral anticipation and 6X AL controls had much higher activity and temperature measurements in the dark period. Although the correlations were positive in all but one case, none of the r^2^ values exceeded 0.4. Thus preprandial temperature was not well correlated to activity, showing that behavioral anticipation can occur in the absence of temperature entrainment.

### Eighteen-hour Interval Feeding Schedule

**Figure 6:**
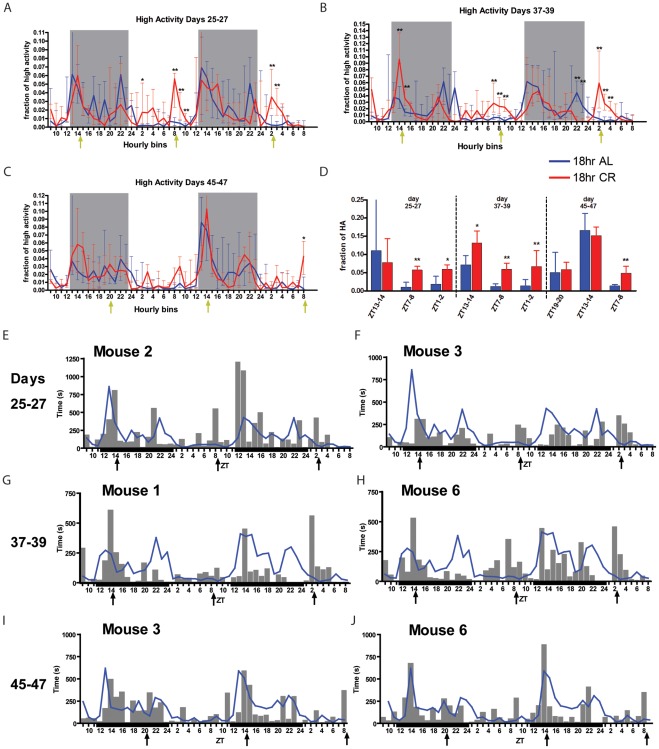
18 h interval feeding schedule. (A) Normalized high activity median +/− IQR profiles during the 48 h recording during days 25–27 for 18 h interval feeding. (B) Normalized high activity profiles for days 37–39. (C) Normalized high activity profiles on days 45–47. Yellow arrows correspond to feeding events. (D) Fraction of high activity during the h preceding feeding events for days 25–27, 37−39, and 45–47. (E–J) Individual high activity mouse data with mean AL values indicated by a blue line for (E) mouse #2 on days 25–27, (F) mouse #3 on days 25–27, (G) mouse #1 on days 37–39, (H) mouse #6 on days 37–39, (I) mouse #3 on days 45–47, and (J) mouse #6 on days 45–47. All statistics were performed using Mann-Whitney * denotes p<0.05, ** denotes p<0.01. n = 6 for AL and n = 6 for CR at all time points.

We next used an 18 h feeding schedule to determine whether mice can anticipate food when meals are provided at a fixed non-circadian interval (Figure 1). 18 h CR mice were fed 45% CR per meal (corresponding to 60% CR per 24 h) and AL control mice received a feeding event at the same times. As expected, during the first two days of the schedule, the activity levels of 18 h AL and CR mice were very similar (Figure S7). On days 25–27, the 18h CR mice showed increased activity prior to consecutive meals at ZT8 and ZT2, both in the light period (Figure 6A, D). On days 37–39, 18 h CR mice showed significant peaks of high activity preceding the ZT 14, 9, and 2 (Figure 6B, D). On days 45–47, pre-meal activity was not elevated above controls at ZT 20 or 14 but there was a notable increase at ZT 8, which occurred during the light period (Figure 6C–D). Normalized high activity during the 2h preceding mealtimes, expressed as a fraction of the daily total, was significantly greater in the 18 h CR group for more than a third of all feedings (8 of 21) and more than half (6 of 11) light period feeding events after day 25 of the experiment (Figure S7).

As with our other interval feeding studies described above, we examined behavioral data from individual mice to determine if sequential meals cycles were being predicted accurately by individual mice and which, if any, were predicted with consistency for a particular mouse (Figure 6E–J; Figure S8). Examining the feeding schedule that was best anticipated (ZT 14, 8 and 2) on days 25–27 for mice #2 and #3 suggested that each of these mice showed FAA for both of the light period feedings (Figure 6E–F). On days 37–39, where feedings were again delivered at ZT 14, 8, and 2, mice # 1 and #6 anticipated the dark cycle feeding at ZT 14 (Figure 6G–H). While mouse #1 did not anticipate the following light period feeding at ZT8, mouse # 6 showed an impressive peak of activity just preceding the ZT 8 feeding and even the following ZT 2 feeding 18 hours later, demonstrating FAA for three successive 18 h interval feedings (Figure 6H). On days 45–47, where there are two consecutive dark period feedings at ZT 20 and 14 followed by a light cycle feeding at ZT 8, mice #3 and #6 both anticipated the ZT 8 feeding but only mouse 6 showed anticipation of a dark period feeding (Figure 6I–J). From these examples, it appears that some mice are capable of predicting 18 h interval feedings but did not do so consistently to every feeding. In fact, most individual mice on an 18 h CR feeding schedule did not anticipate scheduled meal deliveries. Using our cutoffs, CR mice anticipated an average of 2.5 of the 3 meals for days 17–19 (vs. 0.83 for CR mice; p  = 0.003), and 1.83 of the 3 meals for days 25–27 (vs. 1.33 for CR mice; p = 0.11); however, in both cases one of the FTs is soon after the transition from the light- to dark-cycle, for which a sharp increase in activity may be explained by the light-entrained clock. This is reflected in the relatively high average count of ∼1 anticipated FT for AL mice.

### Concurrent Long and Short Interval Feeding Schedule

In a final experiment, we examined whether mice can express food anticipation at both circadian and sub-hourly intervals concurrently. Mice (n = 6) received 60% CR meals in six feedings at 30 min intervals from ZT 4.5 – 7.5 for 22 days (termed “LS CR” for long-short interval). An LS AL control group (n = 6 mice) received food in excess at each mealtime. To pre-adapt to restricted feeding, the LS-CR group was fed one 60% CR meal daily at ZT 8 for 7 days. For the next 21 days food was provided in 6 meals of 10% CR, at ZT4.5, 5, 5.5, 6, 6.5 and 7.

**Figure 7:**
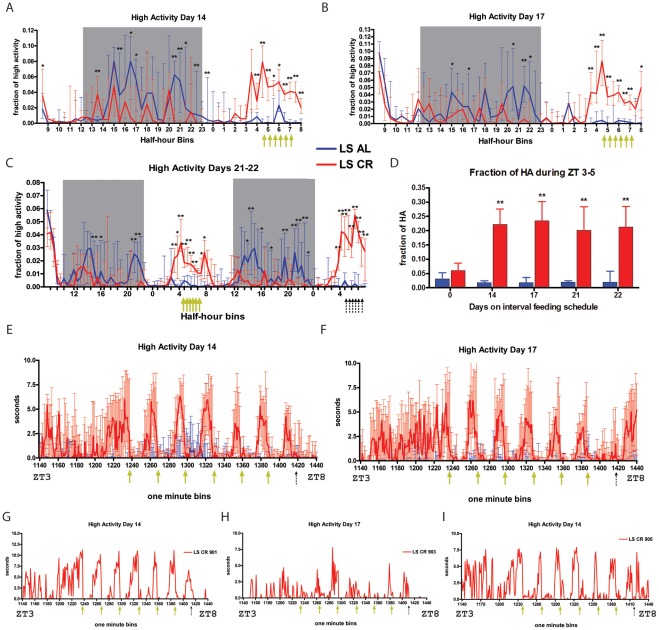
Short interval feeding schedule. (A) Fraction of median +/− IQR normalized high activity for AL and CR mice on day 14 of LS CR, corresponding to the 21^st^ day of 60% CR. (Day -7 is the first day of CR feeding, which occurred as a single feeding event at ZT 8, then the mice were switched to being fed 6 small meals at 30 min intervals beginning at ZT 4.5). (B) Fraction of high activity for AL and CR mice on day 17 (24 total days of 60% CR). (C) Days 21–22 of LS interval feeding (day 28–29 of CR). The yellow arrows indicate the 6 feeding times, whereas the 6 black dashed arrows indicate the expected time of feedings, which were omitted, on day 22 of LS feeding schedule. (D) Fraction of high activity in the two h preceding the first meal delivery for AL and CR mice on days 0, 14, 17, 21, and 22 of LS interval feeding. (E–F) Group median +/− IQR data for high activity shown in one min bins for (E) day 14 and (F) day 17. Min 1140 corresponds to ZT 3 and min 1140 corresponds to ZT 8. Blue line indicated median AL data. (G–I) Individual data for 30 min scheduled CR feeding; (G) s of high activity for CR mouse 901 from ZT3-8 on day 14 of LS interval feeding, (H) s of high activity for CR mouse 903 from ZT3-8 on day 17 of LS interval, and (I) s of high activity for CR mouse 905 from ZT3–8 on day 14 of LS interval feeding. n = 6 for both LS AL and LS CR at all time points.

Group waveforms of data plotted in 30 min time bins reveal a marked increase of high activity beginning ∼ 1.5 h prior to the first meal at ZT4.5 (Figure 7A–C). This was significantly different from the AL group by day 3 of the schedule, and reached an asymptote by day 7 (Figure 7D and Figure S9). Activity remained elevated throughout the 3-h daily feeding window when food was provided every 30 mins, and also on the food deprivation day, day 22, when no feedings occurred (Figure 7C, D).

To determine whether the CR mice fed at short intervals were showing bouts of anticipatory activity on a scale of mins, we then analyzed the high activity data in 1-min bins (Figure 7E–F). On day 0 (day 7 of CR but day 1 of LS CR feeding schedule) peaks of activity were evident after feeding events, but with no consistent periodicity (Figure S10). By day 7 of LS CR, a 30 min cycle of activity was clearly evident, with activity rising about 15 min prior to each feeding event (Figure S10). These activity peaks were sharper on days 14 and 17 of the LS CR schedule (Figure 7E–F). AL control mice show little to no activity during these feeding intervals, confirming that activity is not due to a disturbance caused by the feeder.

The video recordings were continued for almost one half h beyond the sixth and final daily feeding event, to determine if anticipation was linked to a specific phase of a 24 h clock when meals reliably occurred, or whether it reflects a 30 min timing process that continues for at least one cycle after the last feeding. On day 14, a peak of activity was evident ∼30 min after the last food drop, although no food was ever provided at that time (Figure 7E). On day 17 of the feeding schedule, there was no clear peak of activity during the 30 min interval after the 6th feeding, but a robust peak was evident ∼20 mins late (Figure 7F).

Data from individual mice indicates that there was substantial meal anticipation of all 6 LS meals, and in some cases a 7th peak after the last meal (Figure 7G–I). Using our cutoffs, CR mice anticipated an average of 5.5 of the 6 meals on day 14 (vs. 0.17 for AL mice; p = 0.001), and an average of 4.3 of the 6 meals of day 17 (vs. 1.0 for AL mice; p = 0.002). Interestingly, for mouse 901 on day 14 (Figure 7G) and mouse 903 on day 17 (Figure 7H), the 7th peak of activity subsides even though there is no feeding event whereas for mouse 905 on day 14 the activity does not subside for long and spikes again after the expected feeding time (Figure 7I). Additional individual mouse data for LS feeding is shown in Figure S11. On the food deprivation day, day 22, there was no pattern of 30 min cycles during the expected feeding time range (Figure S12). To investigate the possibility that the CR mice were exhibiting continuous FAA throughout the feeding periods that was being disrupted only by physically eating, we quantified meal duration by manually scoring the amount of time each mouse spent eating after food was delivered on days 17 and 21 of LS interval feeding. We found that each meal lasted, on average, between 12 and 15 min, with the last meal taking longer (Figure S13).

## Discussion

Food restricted rats and mice fed once a day at a fixed time become active during the hours immediately preceding mealtime. There is a wide range of conceptually distinct mechanisms that could yield daily meal timing. Experimental analysis of food anticipation in rats has produced an array of findings consistent with the concept of a food-entrainable circadian oscillator anatomically distinct from the light-entrained master circadian clock in the mammalian SCN [3]. Rats can also readily anticipate two daily meals, but reportedly not three, suggesting a dual oscillator structure, with dissociable FEOs capable of tracking two, but not three daily meals independently. In the present study, we sought to determine the limits of anticipatory behavior in the mouse, which is the species of choice for molecular genetic analysis of food anticipatory rhythms, despite a gap of knowledge needed to confirm canonical circadian properties of anticipatory rhythms in this species. We tested C57BL/6J male mice of approximately the same age using an array of feeding interval schedules but retaining 60% CR across all of these experiments. While this ensures internal consistency within our study, it hinders comparison to other studies that used different methods of food restriction and activity measurement.

We first confirmed that mice can anticipate two daily meals, quantified using a semi-automated behavior recognition system. Contrary to results obtained for rats [31], we then found evidence for anticipation of more than 2 daily meals in mice with access to 3 or 6 daily meals, provided at intervals of 8 h or 4 h, respectively. In group data, anticipation was visible for all three meals on the 3X CR schedule, and for up to 5 meals on the 6X CR schedule. Group data could be misleading, if different mice anticipate no more than 2 daily meals, but at different mealtimes. Careful inspection of the individual activity records confirms that individual mice can indeed anticipate meals at 4 h and 8 h intervals, albeit with variable success. The meals anticipated by individual mice were not always consistent across weekly video recordings or between mice.

Entrainment of a non-circadian oscillator is suggested by the evidence for anticipation of meals at 18 h intervals. Anticipation of meals at intervals of 14, 16 or 20 h has been reported in rats [24], [26], but most other studies have not observed anticipation at intervals below 23 h [1], [9], [18]. This may be related to the experimental procedures, as studies reporting anticipation in rats at intervals below 23 h measured operant responding for food [24], [26], whereas those failing to observe anticipation measured wheel running. Different behaviors may emerge at different times prior to a scheduled meal, and it is possible that wheel running emerges later than does operant nose poking in rats, or the ‘high-activity’ measures quantified in the present study. It may also be important that on the 18 h feeding schedule employed in the present study, meals fell at the same time of day (ZT2, 8, 14 or 20) once every fourth day. If rodents can discriminate the phase of a circadian clock, it is conceivable that they can learn that food recurs each day at one or more of 4 possible circadian phases, and that this probability is sufficient to support a temporal discrimination. This would explain why the evidence for 18 h anticipation in our mice is clearly weaker than it is for 24 h anticipation. Additional experiments, using constant darkness and/or wheel running as a metric of FAA, will be needed to confirm anticipation of 18 h and other long, non-circadian intervals. Even in the case of 4, 8, and 12 h meal feeding schedules it is important to note that meal recurred at the same time each and every day, providing a strong circadian element to these experiments. One the other hand, one bold interpretation of these results is that the neural substrate of FAA (often referred to as the food entrainable oscillator) is not a circadian entity, though it can cooperate with circadian oscillators under normal physiological conditions. This conjecture is in agreement with Storch and Weitz's study of FAA in circadian clock mutant mice, where they found that Bmal1 mutant mice can anticipate daily feeding events with high fidelity despite lacking a functional circadian system [32]. Perhaps such a non-circadian oscillator would make use of transcriptional/translational machinery with an operating range broader than that of the core circadian clock.

The most robust anticipation observed in the present study was in response to the 6 meal ‘long-short’ interval schedule in which single food pellets were delivered at 30 min intervals near the middle of the light period. Mice under this schedule exhibited prominent anticipation of the daily event, as well as anticipation of each of the individual meals. The 30 min rhythm induced by this procedure reflects in part a pause in activity associated with eating. However, the appearance of a 7th discrete bout of activity of similar duration following the 6th and last meal of each session is evidence for a 30 min anticipatory timing process. This timing process was actuated each day by presentation of the first food pellet, as no 30 min rhythm was evident during the food deprivation day, despite the marked increase of activity during the expected 2.5 h feeding window. This study presents a clear case of interaction between circadian and interval timing mechanisms in response to scheduled feeding events. It will be important in future studies to test FAA across these long and short intervals simultaneously in mutant mice that are failing to show FAA across circadian time windows.

No mouse exhibited clear anticipatory activity to every meal on every recording day, especially in the 3- and 6-meal schedules. It is possible that expression of anticipatory activity at some mealtimes on some days is constrained by competing processes that serve sleep homeostasis or energy conservation. Similar processes may explain the failure of rats to anticipate more than 2 of 3 daily meals, especially given that anticipation in those studies was measured in wheel running, an energetically costly behavior [18]. Mice in the present study did not have access to wheels, which may have permitted expression of anticipatory activity at more mealtimes. Consistent with this interpretation, in pilot studies we have observed stable anticipation of 3 daily meals in SCN-ablated rats, using a food-bin measure of activity instead of running wheels (R.E. Mistlberger and E.G. Marchant, unpublished results). This is consistent with studies by Escobar and colleagues, who showed that the SCN is inhibited in rats on a consistent restricted feeding schedule but not in rats fed at variable times [33]. Interestingly, studies of the expression of *period* genes in the brain have shown that circadian genes expressed in structures such as the dorsal medial hypothalamus can show entrainment in the absence of behavioral anticipatory activity [34], [35]. The limbic system and in particular, the dorsal striatum, shows robust rhythmic expression of circadian components [36] and deserves further attention for it potential role as a mediator of FAA.

Rats and mice restricted to a single daily meal in the middle of the light period exhibit marked alterations in body temperature in parallel with activity. Body temperature spikes at mealtime, declines to below normal values during the late night and early light periods, and then rises during the hours immediately preceding mealtime [37]. The strong correlation between activity and temperature during the pre-meal hours suggests regulation by a common timing mechanism. In our 6 meal, 4 h interval study, we observed a lack of correlation between prepradial activity and temperature, with activity rising prior to some mealtimes without a significant anticipatory rise in body temperature. This suggests that the timing mechanism responsible for behavioral anticipation of meals at 4-h intervals does not directly drive body temperature rhythms. The result also reinforces the argument that core body temperature is not a good substitute for direct measures of behavior [38], [39].

Collectively, our results show that the effective time-keeping capabilities of mice are quite remarkable. These results establish a video-based phenotyping paradigm for experimental analysis of food anticipatory behavior in mice fed at intervals in the mins, hours and circadian range. This model can be exploited to determine the extent to which behavioral timing across a wide range of intervals relies on common or distinct neural and molecular mechanisms.

## Materials and Methods

### Ethics Statement

These experiments were approved by the Caltech Institutional Animal Care Committee under protocol #1567. Every effort was made to minimize pain, distress, and the overall number of animals used in this study.

### Behavioral analysis

Video-based activity data was analyzed using HomeCageScan 3.0 [29], [40], [41]; behavioral definitions were as described previously [29], [42]. High intensity activity was defined as walking, jumping, rearing, and hanging behaviors. There are several other activity related behaviors that are not include in “high activity”, including ‘sniffing’, ‘turning’, ‘stretching’, ‘food bin entry’, ‘unknown’, ‘grooming’ and ‘chewing’. Activity data were accumulated in 30 or 60 min time bins and evaluated for statistically significant changes using non-parametric tests such as the Mann-Whitney Test using GraphPad InStat. All graphs were made with GraphPad Prism 4; medians are reported +/− interquartile ranges. Sample sizes are indicated in the Figure Legends.

A mouse was classified as anticipating a meal if the following two criteria were met: (1) its activity increased at least 50% during the interval preceding the feeding, with interval length 4 hours for 2–4X daily feeding; 2 hours for 6X daily feeding; or 15 mins for the 30 min feeding schedule; (2) its mean activity in the second half of this interval was at least 25% higher than the median activity for the entire population of mice (matched for AL or CR) across the entire LC or DC (matched for the FT). For the 30 min feeding schedule with 1 min time bins, the population medians were based on the 5 h up to and including the feedings (as shown in Fig 7E and Fig 7F) rather than the entire LC. Prior to least-squares fitting of slopes and means of activity levels in these intervals, the per-time-bin activity levels were log_2_-transformed, after adding 5% of the maximum value attained by any mouse in any time bin to avoid logs of zero. Then a 50% increase in activity during the pre-feeding interval corresponds to an increase of log_2_(1.5)  = 0.58 in the fitted line during this interval. Note that using these criteria, individual AL mice were on average classified as anticipating 10–15% of meals. To control for this baseline rate, one-tailed Wilcoxon rank-sum tests were used (with the R package coin, to handle ties) to compare the counts of meals anticipated by CR mice to the counts of meals anticipated by AL mice.

### Automatic feeding apparatus

All mice were exposed to feeders at least four days prior to the beginning of the CR schedule. A comparison between a group of CR mice hand-fed once daily (n = 4) and a group of CR mice fed by automatic feeder (n = 4) once daily revealed similar magnitudes and distribution of all behaviors (such as walking, jumping, and rearing) with the exception of an increase in the total time hanging (but no change in the distribution of hanging) in mice fed by the automatic feeders. Possibly this increased hanging was an attempt to reach the inaccessible food that was stocked in the feeder overhead.

Automatic feeders were constructed at Caltech. Food pellets were stored in a plastic tube suspended above the wire rack. At the scheduled time, a piston would advance, pushing the pellet through a hole to drop it into the wire rack below. A laser sensor is disrupted by the passage of a food pellet and this stops the advancing of the plunger. The automatic feeders were accurate to within 5 min and were reset at least once every two to three days. There were almost no instances of failures of these feeders to deliver food; rather they occasionally distributed two pieces of food when the pellets were cut into small pieces in the case of 6X CR feeding. We minimized these “double drop” events in 6X feeding experiments by restocking feeders daily, which prevented the uneven clustering of pellets in the plastic tube.

### Mouse strains and feeding conditions

All mice were male C57BL/6J mice aged 10–14 weeks at the onset of the experiment (Jackson Labs West) except for the mice used for temperature monitoring experiments, which were 6 months old. These mice were entrained to a 13:11 LD cycle and single-housed for 4–6 days with AL access to food (Laboratory Rodent Chow Type 5001) and water prior to being placed on special feeding protocols. Daily food intake was measured over a 48 h period beginning two or three days after single-housing. For all experiments, the mass of food provided to CR mice in any 24 h period was equal to 60% of the average daily intake during AL food availability, which was measured over a two day period in the week prior to initiating CR. In all cases, mice consumed the entirety of each meal (i.e., we never observed left over pellets in the food bins). The size of CR meals was not adjusted across the experiment. Sample sizes ranged from n = 6 to 18 for CR and AL controls and the specific sample size for each experiment is listed in the figure legends.

Automatic feeder device failure resulting in absent or mistimed feeding events led us to remove the affected CR mice from the study. Infrequent double drops and missed meals occurred for some mice subjected to the 6X CR. Feeders were restocked every 3 days for 2x mice, 2 days for 3x mice, daily for 6x mice, and once every two to four days for 18 h feeding-interval mice. Control mice received feeding events at the same time as the CR mice; the amount of AL food in the food bin was minimized in order to provide similar mechanical, auditory, and olfactory stimulation for both CR and AL-fed mice.

For the final recording of the 6X mice, the mice were recorded for 48 h to test the robustness of the putative entrainment to food presentation. The first 24 h only differed from a normal recording in that ‘false’ feeding events, marked by delivery of an inedible plastic pellet, replaced the third (ZT  = 16) and fifth (ZT  = 24) feeding events. During the second 24 h period the mice were fasted in constant darkness.

For 18h feeding interval experiments, the CR group (n = 6) received 45% of its daily food intake for each meal (such that it received 60% of its initial daily food intake during any 24 h period) while the AL group had constant food availability in addition to the food supplied by the feeders. Due to a shift of the feeding intervals in relation to the light schedule, feeding events occurred in the following sequence across a three day period: ZT 8, ZT 2, ZT 20, ZT 14, and ZT 9.

For L-S interval feeding experiments, due to problems with the timer in the feeding apparatus, mice in this experiment were first subjected to 7 days of 60% CR fed in one single meal daily at ZT 8. Then once interval feeding began, the first feeding usually occurred about 35–40 min after ZT 4, instead of at ZT5, which was the intended target time. Each subsequent feeding then occurred almost exactly 30 min after the first feeding event.

For measurement of core body temperature, mice were implanted with “ibuttons” (Maxim-Dallas) in the peritoneal cavity as described previously [43].

## Supporting Information

Figure S1 **12 hour interval feeding schedule.** The amount of normalized high activity in the 2 h preceding each feeding on days 7, 14, 21, 28, 35, and 42. Statistical significance was determined using the Mann-Whitney Test with asterisks denoting * = p<0.05, **  = p<0.01, *** = p<0.001. n = 5−8 mice for both 2X AL and 2X CR at all time points.
(PDF)

Figure S2 **Group high activity data for 12 hour interval feeding for days 7, 21, 28, and 35 (no statistical testing indicated).** Individual mouse high activity data for 12****hour interval CR feeding for days 7, 14, 21, 28, 35, and 42. Blue line indicates mean AL control activity.
(PDF)

Figure S3 **8 hour interval feeding schedule.** The amount of normalized high activity in the 2 h preceding each feeding on days 0, 14, 21, 28, 35, and 42.
(PDF)

Figure S4 **Individual mouse data for 8 hour interval feeding schedule for days 0, 7, 14, 21, 28, 35, and 42.** Blue line indicates mean AL control activity.
(PDF)

Figure S5 4 **hour interval CR feeding schedule.** The amount of normalized high activity in the 1 h preceding each feeding on days 0, 7, 14, 21, 28, 35, and 42–43. The blue line indicates mean AL control activity.
(PDF)

Figure S6 4 **hour interval CR feeding group data for days 0 and 28.** Individual mouse data from 4 hour interval fed CR mice on days 0, 10, 18, 21, 28, 32, 35, 38 and 42–43.
(PDF)

Figure S7 **18 hour interval feeding group median data shown for days 0–2, 25–27, 37–39, 45–47, and 53–54.** The amount of normalized FAA during the 2 hours before each me is graphed in panel F.
(PDF)

Figure S8 **18** **hour interval individual mouse high activity data for days 0–2, 9–11, 17–19, 25–27, 29–31, 33–35, 37–39, 41–43 and 45–47.** The feedings are organized by “type”, where “type A” was when feedings occurred at the beginning of a video recording at ZT 9, then at ZT 2, and finally at ZT 20; days 9, 29, and 41 are shown for type A. “Type B” recordings feedings occurred at ZT 20, then ZT 14, and finally at ZT 8; days 0 (the first day of CR), 33, and 45 are shown. For “type C” recordings, feeding occurred at ZT 14, ZT 9, and ZT 2; days 17, 25, and 37 are shown. Anticipatory activity for 18 h interval CR feedings was most visible in type C recordings, possibly because two of the feedings occur during the light cycle.
(PDF)

Figure S9 **Short interval CR feeding experiment.** (A) Fraction of high activity (in half hour bins) for AL and CR mice on Day −7. Day −7 is the first day of CR feeding, which occurred as a single feeding event at ZT 8. (B) Fraction of high activity for AL and CR mice on day 0, which was the first day of 30 minute interval feeding but the 7th day of 60% CR. The yellow arrows indicate the 6 feeding times (C) Day 14 of LS interval feeding (day 21 of CR). (D) Fraction of high activity for AL and CR mice on day 17 of LS interval feeding (day 24 of CR). (E) Fraction of high activity for AL and CR mice on day 21 of LS interval feeding (day 28 of CR). (F) Fraction of daily high activity for AL and CR mice during ZT 3–5 (2 h preceding feeding) at every recorded day of LS interval feeding. (G) Fraction of high activity for AL and CR mice on days 21 and 22 of LS interval feeding (days 28 and 29 of CR). n = 6 for both LS AL and LS CR at all time points. Yellow arrows represent feeding times and dotted black arrows indicate times at which feeding times would normally have occurred if the 30 minute cycle had continued.
(PDF)

Figure S10 **Anticipation of 30** **minute meal delivery cycles.** (A) High activity data (in seconds) for AL controls and LS CR mice is shown in 1 minute bins from ZT3-8 on Day 0 of LS interval feeding(the 7th day of CR). The x-axis is in minutes. Day 0 is the first day of LS interval feeding but day 7 of CR. (A) High activity data (in seconds) for AL controls and LS CR mice is shown in 1 minute bins from ZT3-8 on Day 0 of LS interval feeding (the 7^th^ day of CR). Day 0 is the first day of LS interval feeding but day 7 of CR. (B) Seconds of high activity for AL and CR mice from ZT3-8 on day 7 of LS interval feeding (day 14 of CR). (C) Seconds of high activity for AL and CR mice from ZT3-8 on day 14 of LS interval feeding (day 21 of CR). (D) Seconds of high activity for AL and CR mice from ZT3-8 on day 17 of LS interval feeding (day 24 of CR). (E) Seconds of high activity data for day 21 and part of day 22. (F) Seconds of high activity for day 22 during the time at which meals were normally scheduled, but were withheld during this experiment. Normal feeding times indicated by dashed arrows.
(PDF)

Figure S11 **Individual LS CR mice high activity data in 1 min bins for days 14, 17, and 21 shown for mouse 901, 903, 905, 907, 909, and 911.**
(PDF)

Figure S12 **Short interval feeding schedule.** Seconds of high activity for LS CR mice 901, 903, 905, 907, 909, and 911 for day 22 when meals were not delivered. The 30 minute intervals when food was normally delivered are indicated by black arrows. Minute 1140 corresponds to ZT 3 and minute 1140 corresponds to ZT 8.
(PDF)

Figure S13 **Quantification of meal duration.** Meal duration was quantified by manually scoring the amount of time each mouse spent eating after food delivery for day 17 and day 21 of LS interval feeding. For day 17, n = 4 mice and for day 21, n = 6 mice.
(PDF)
