# Supplementary figures and images for: Food Anticipatory Activity Behavior of Mice across a Wide Range of Circadian and Non-Circadian Intervals
Source: PLoS One. 2012 May 25;7(5):e37992. doi: 10.1371/journal.pone.0037992 (PMC3360658; doi:10.1371/journal.pone.0037992)

## Fraction of high activity 2 hours before feeding

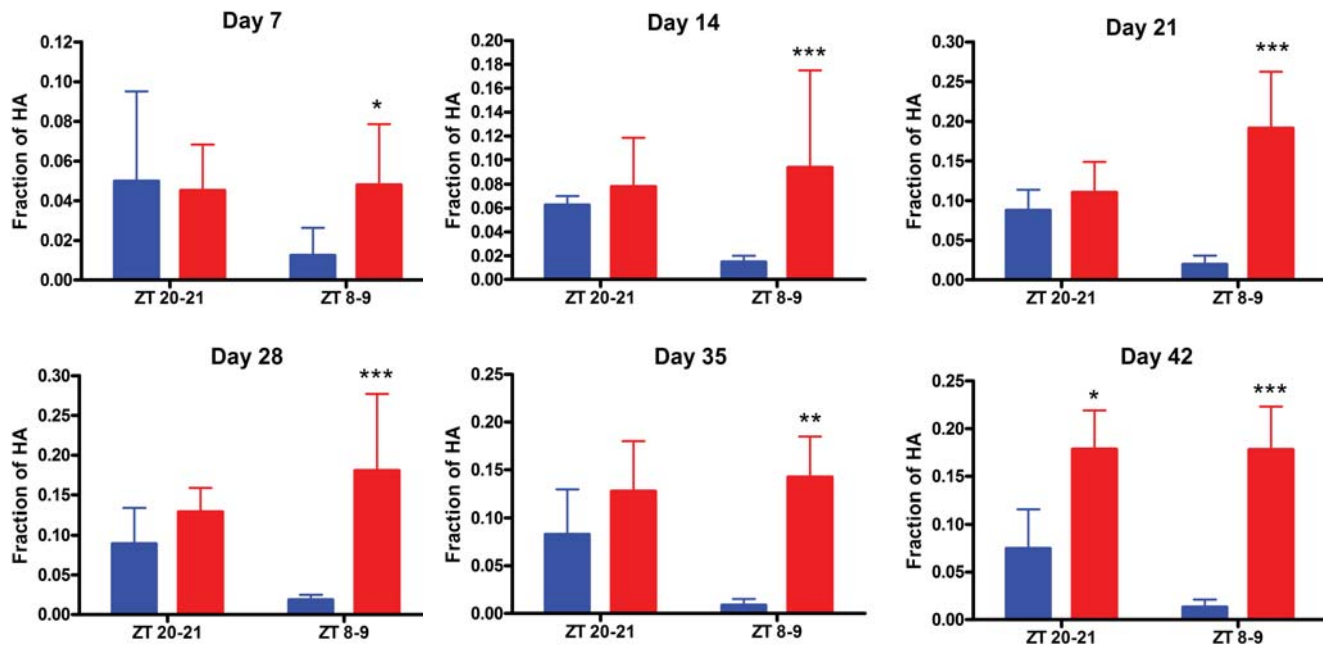

Supplement: Figure S1 — 12 hour interval feeding schedule. The amount of normalized high activity in the 2 h preceding each feeding on days 7, 14, 21, 28, 35, and 42. Statistical significance was determined using the Mann-Whitney Test with asterisks denoting * = p<0.05, ** = p<0.01, *** = p<0.001. n = 5−8 mice for both 2X AL and 2X CR at all time points. (PDF) [file pone.0037992.s001.pdf]

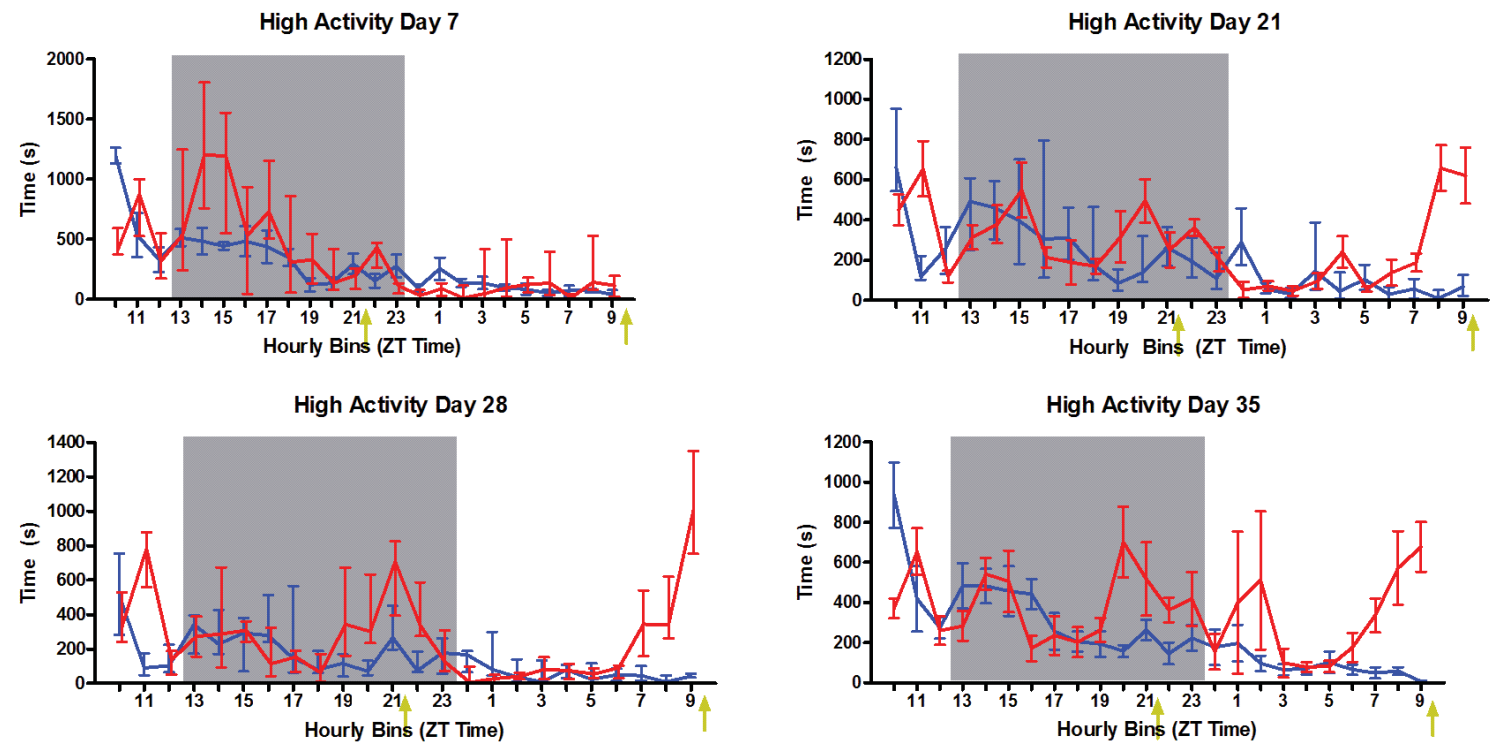

## Day 7

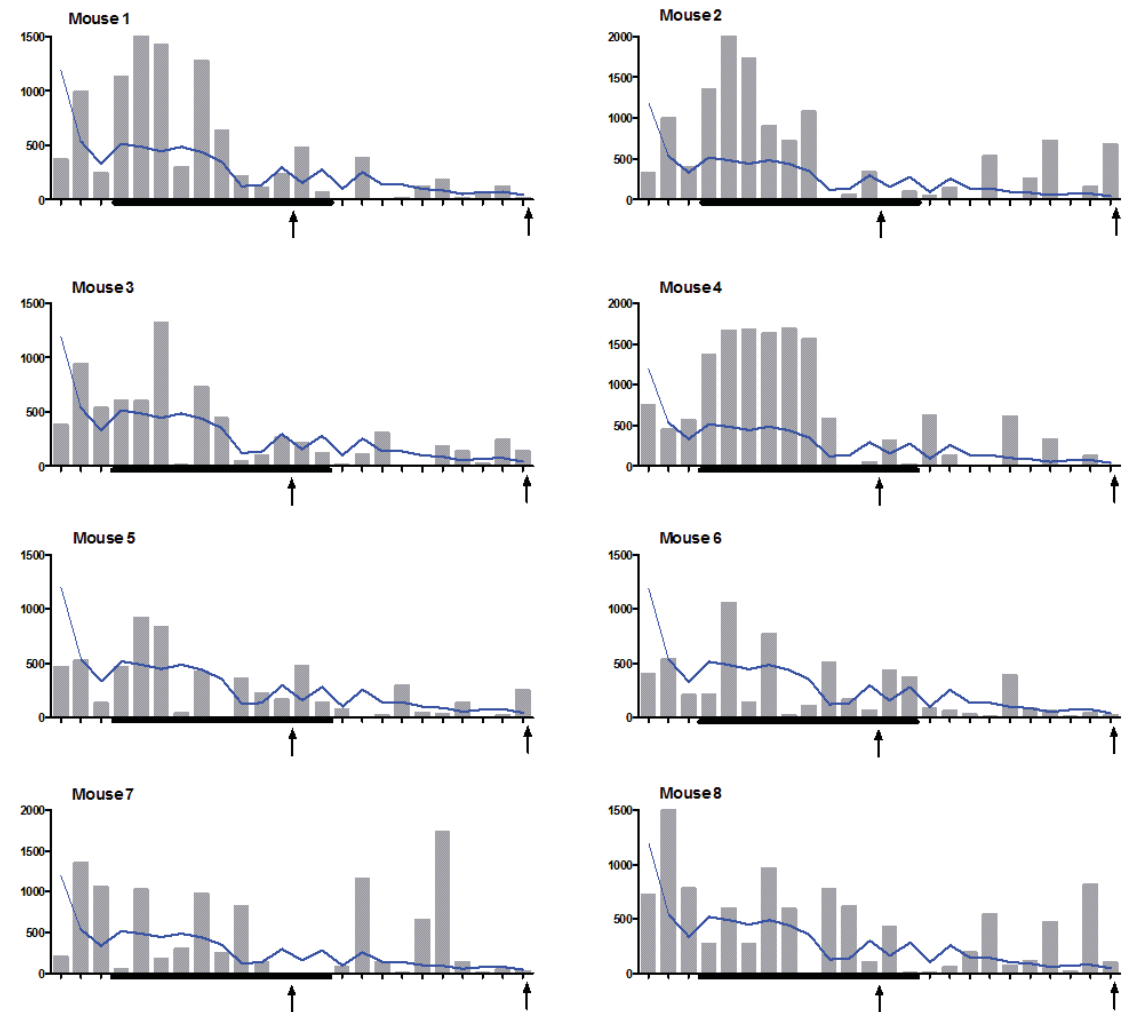

## Day 14

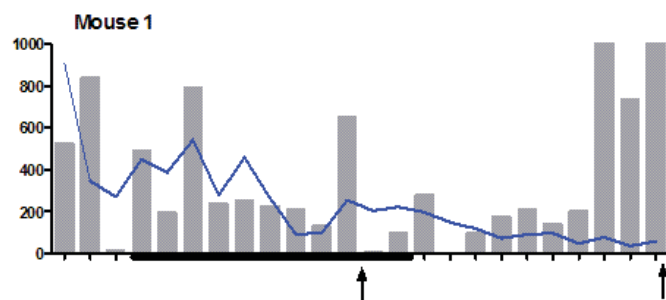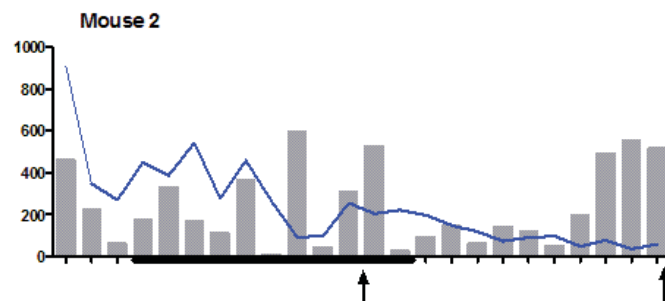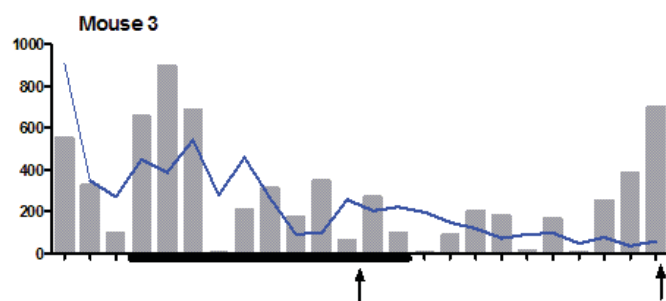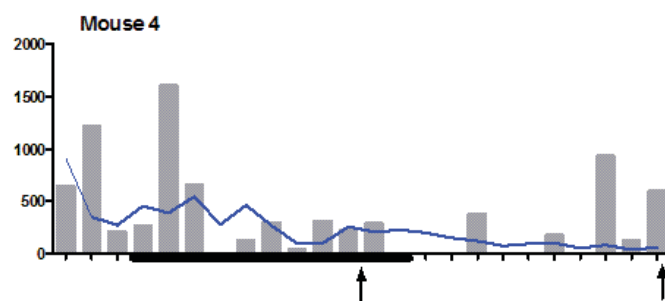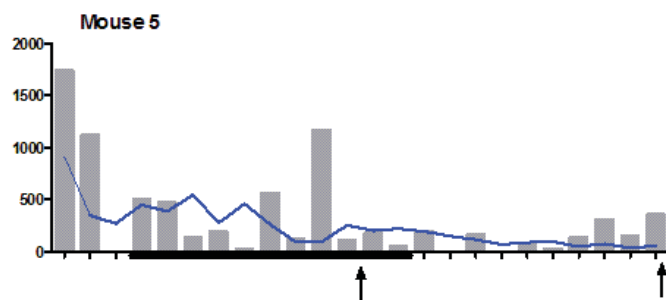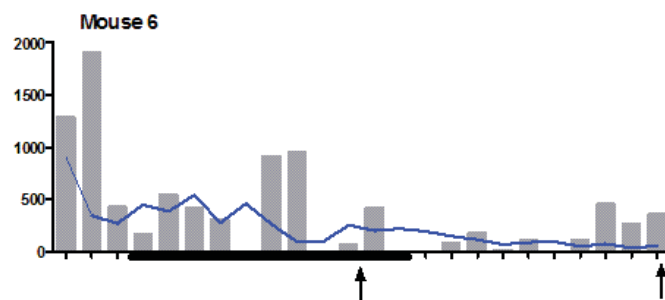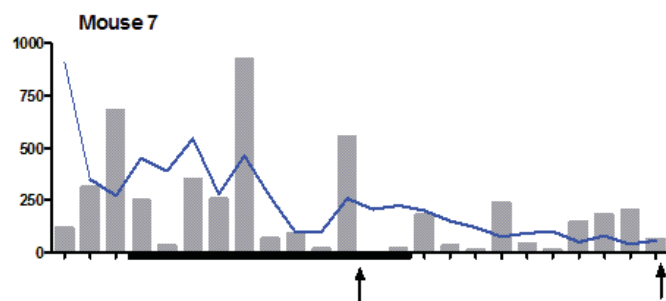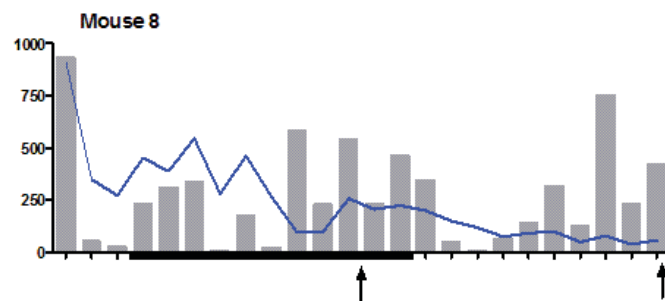

## Day 21

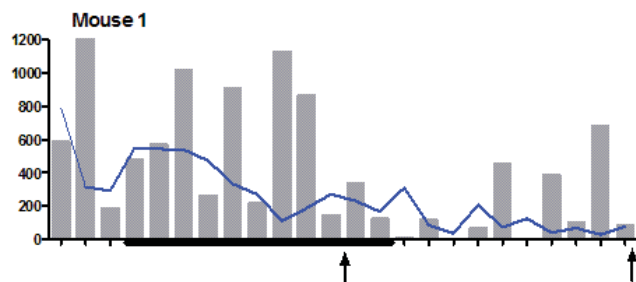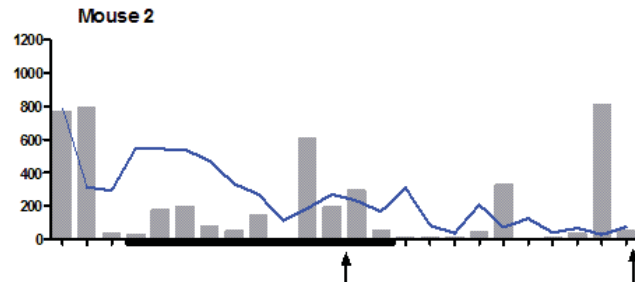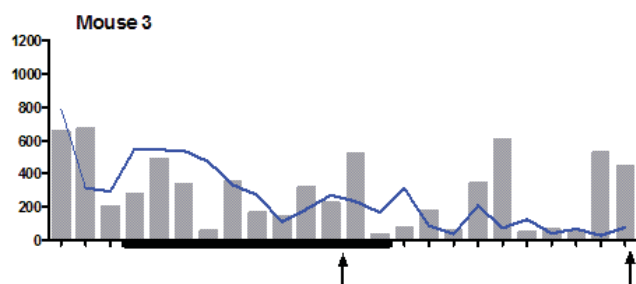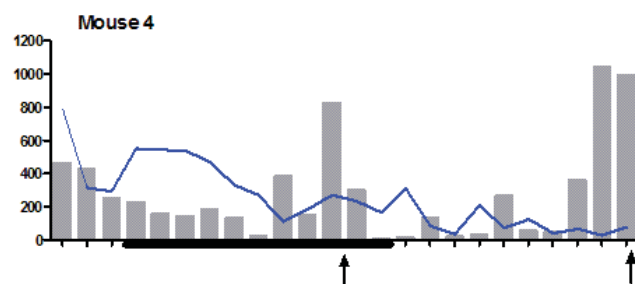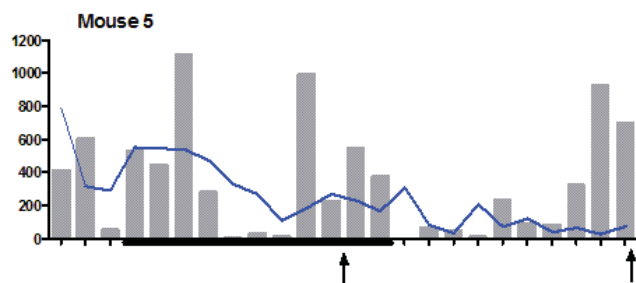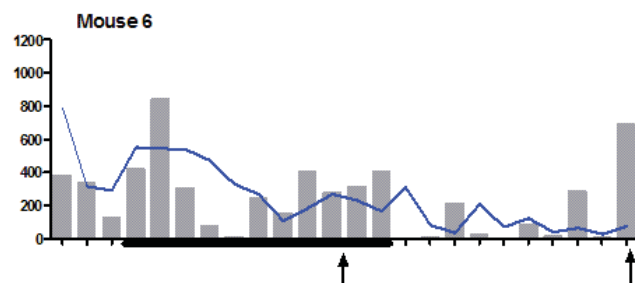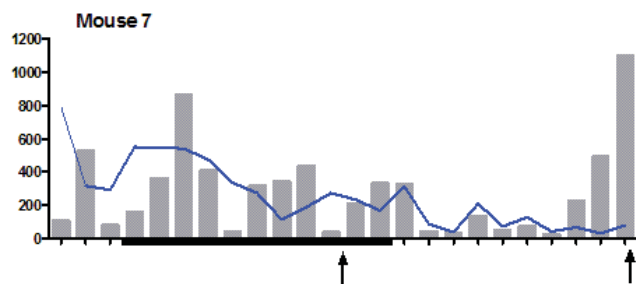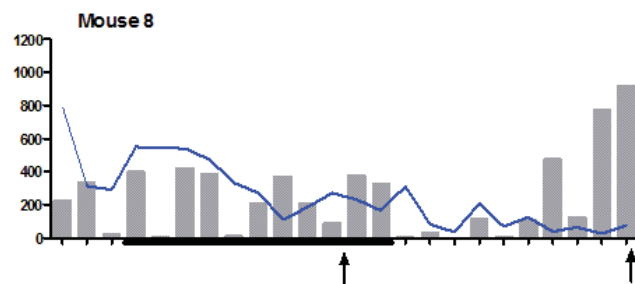

## Day 28

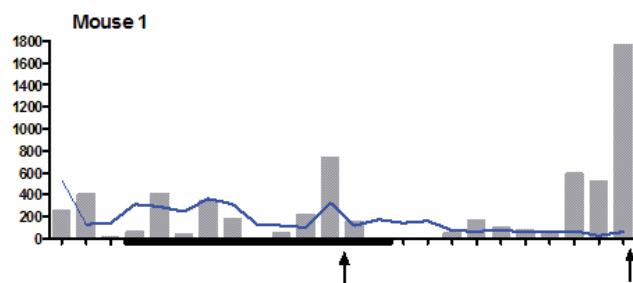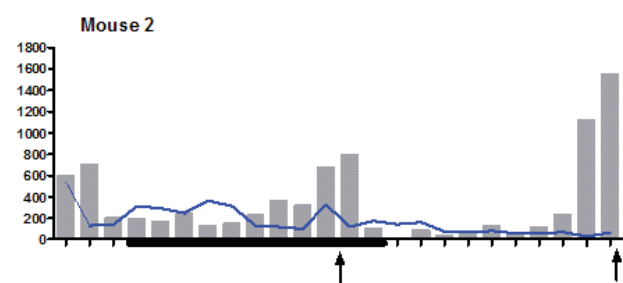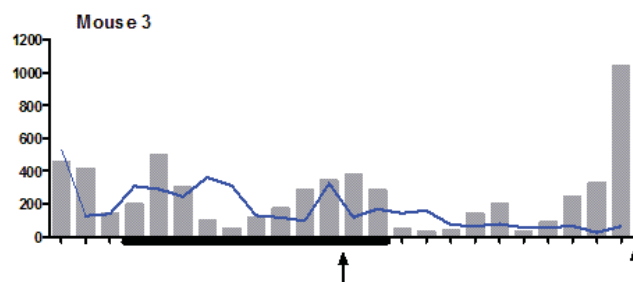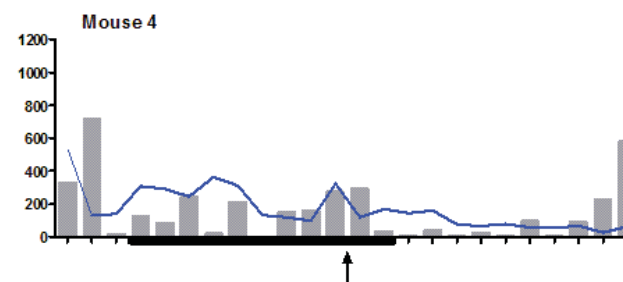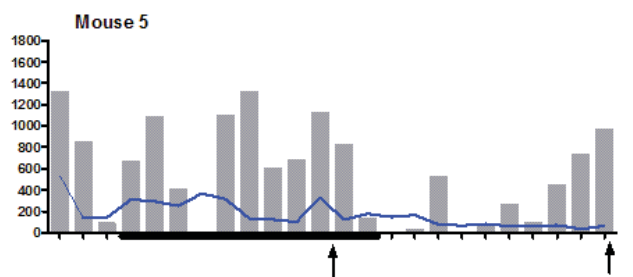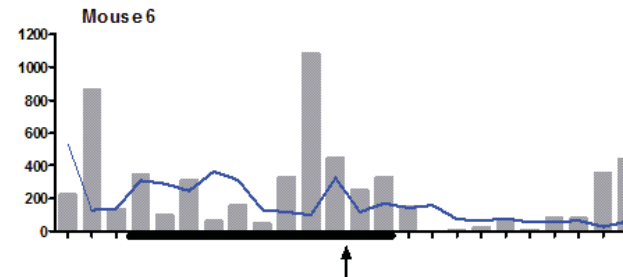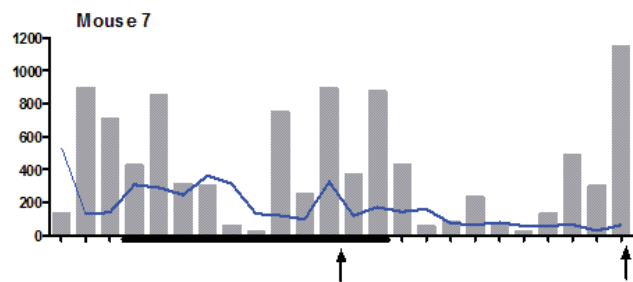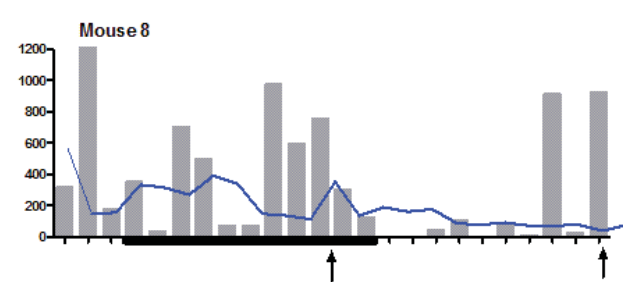

## Day 35

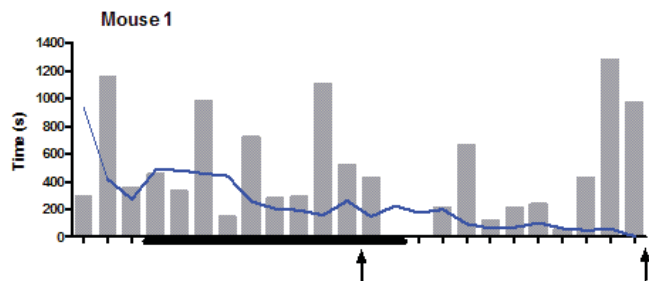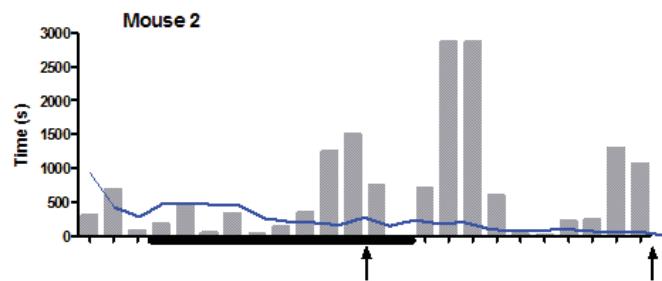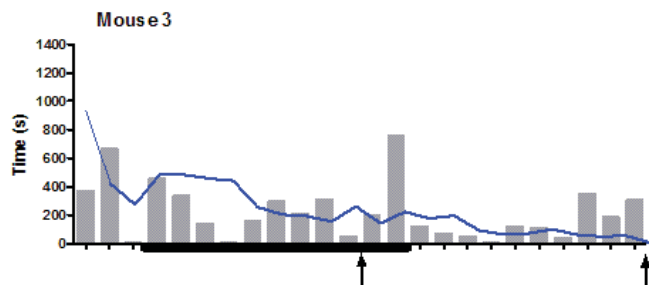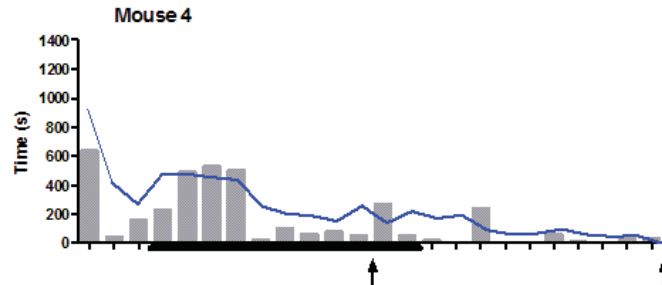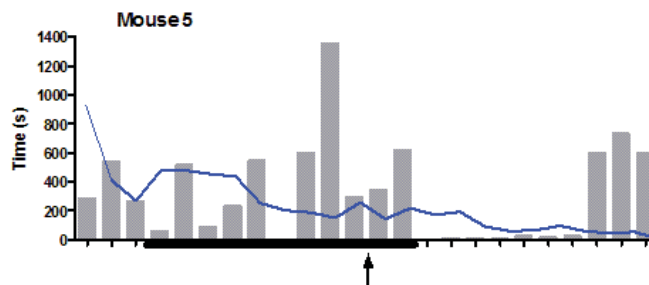

## Day 42

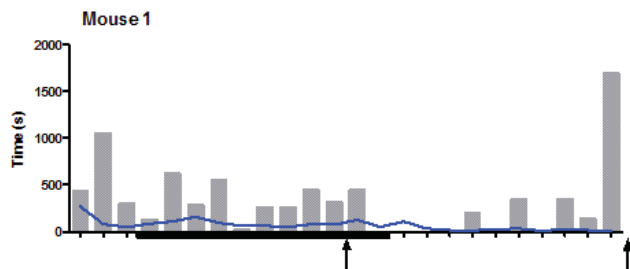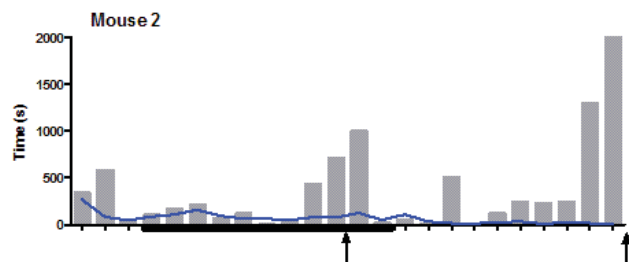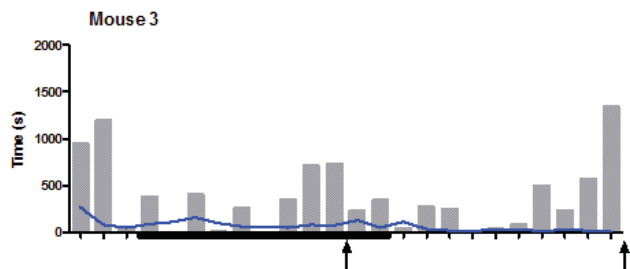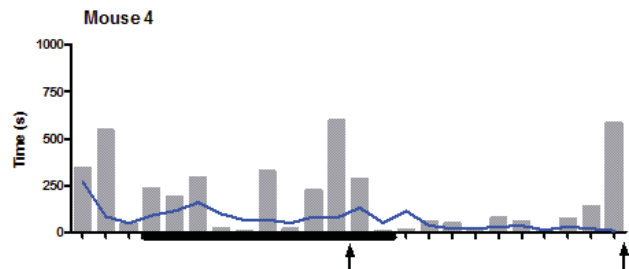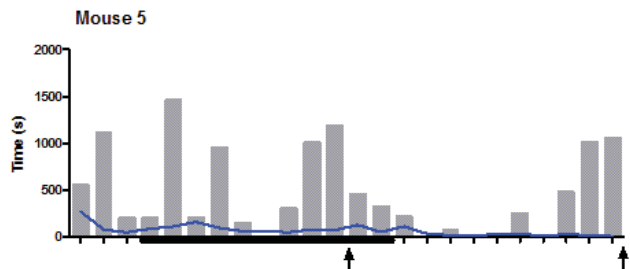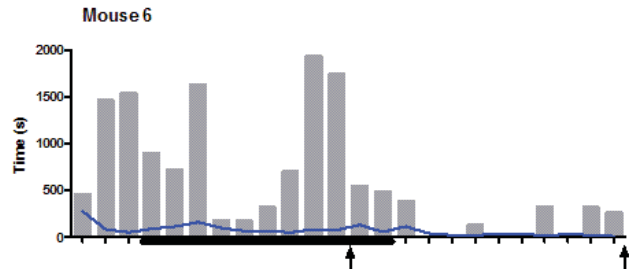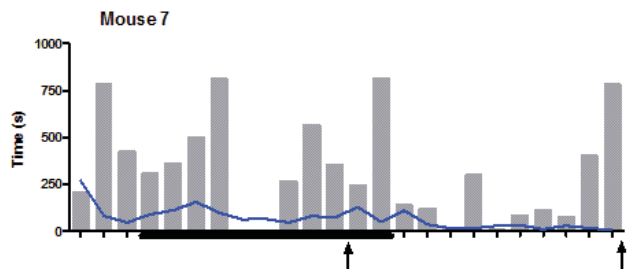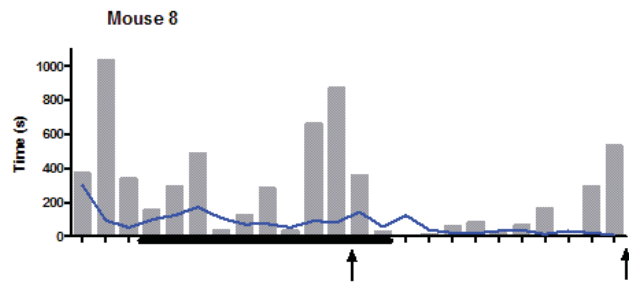

Supplement: Figure S2 — Group high activity data for 12 hour interval feeding for days 7, 21, 28, and 35 (no statistical testing indicated). Individual mouse high activity data for 12hour interval CR feeding for days 7, 14, 21, 28, 35, and 42. Blue line indicates mean AL control activity. (PDF) [file pone.0037992.s002.pdf]

## Fraction of high activity 2 hours before feeding

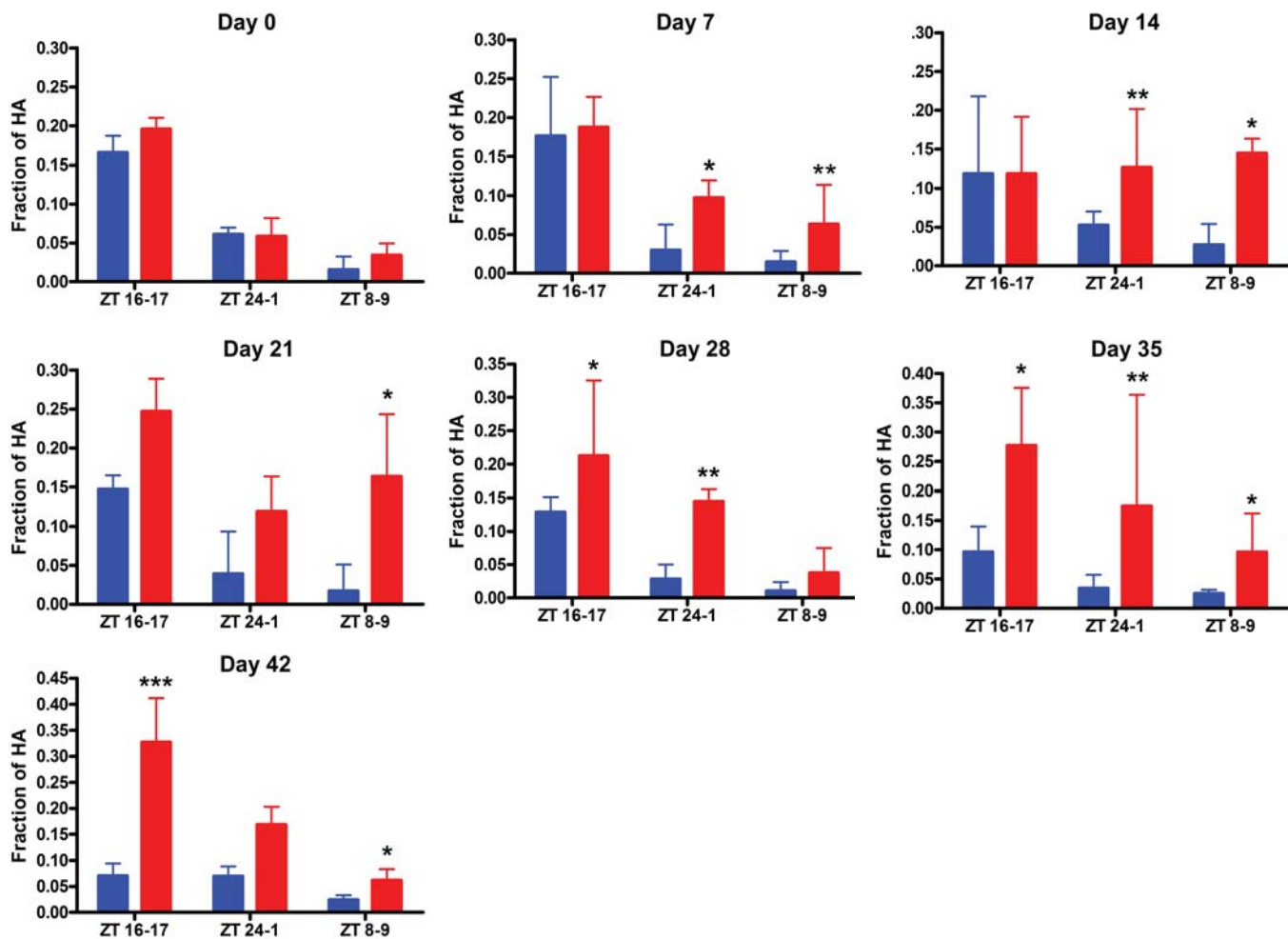

Supplement: Figure S3 — 8 hour interval feeding schedule. The amount of normalized high activity in the 2 h preceding each feeding on days 0, 14, 21, 28, 35, and 42. (PDF) [file pone.0037992.s003.pdf]

## Fraction of high activity 1 hour before feeding

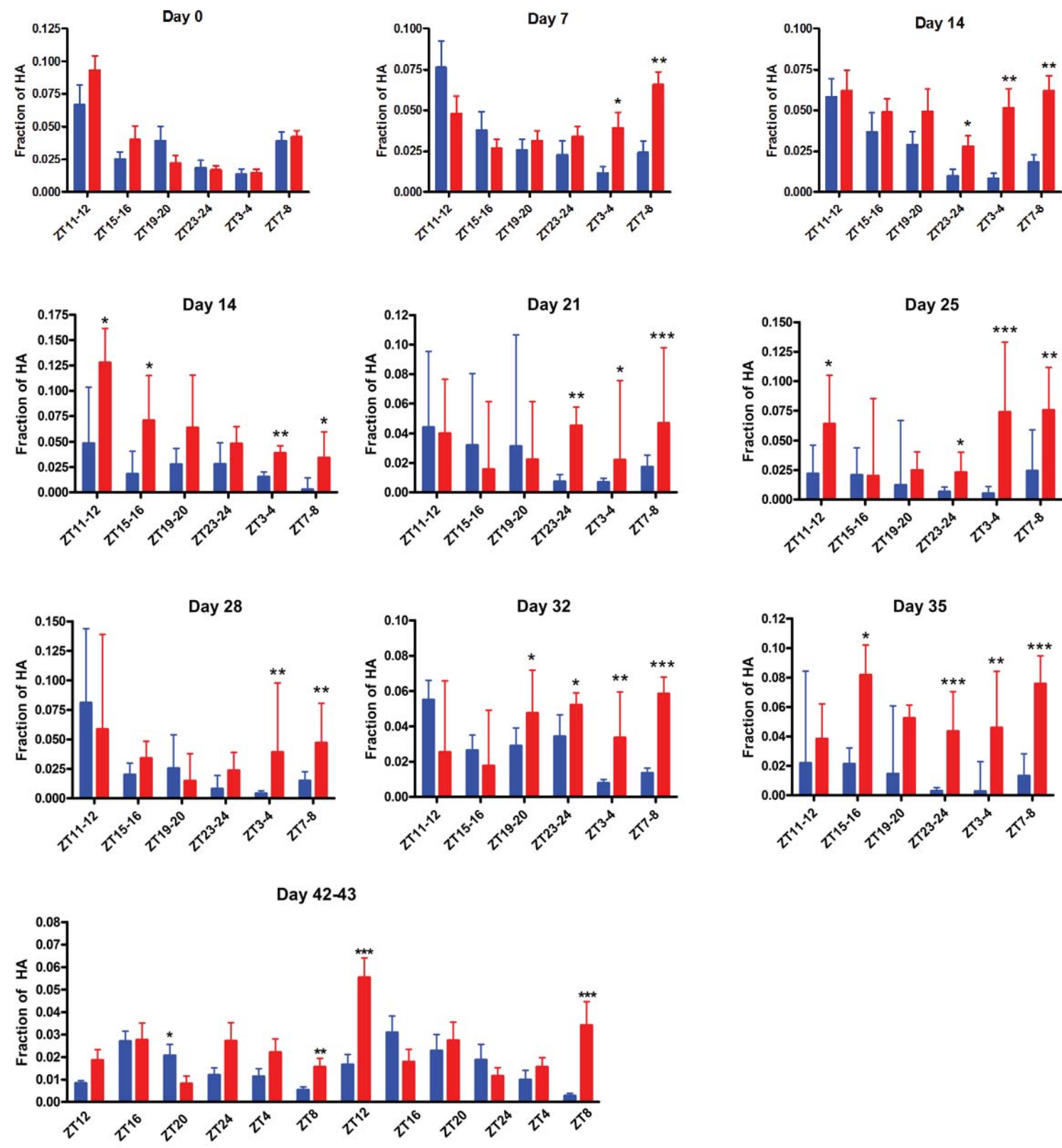

Supplement: Figure S5 — 4 hour interval CR feeding schedule. The amount of normalized high activity in the 1 h preceding each feeding on days 0, 7, 14, 21, 28, 35, and 42–43. The blue line indicates mean AL control activity. (PDF) [file pone.0037992.s005.pdf]

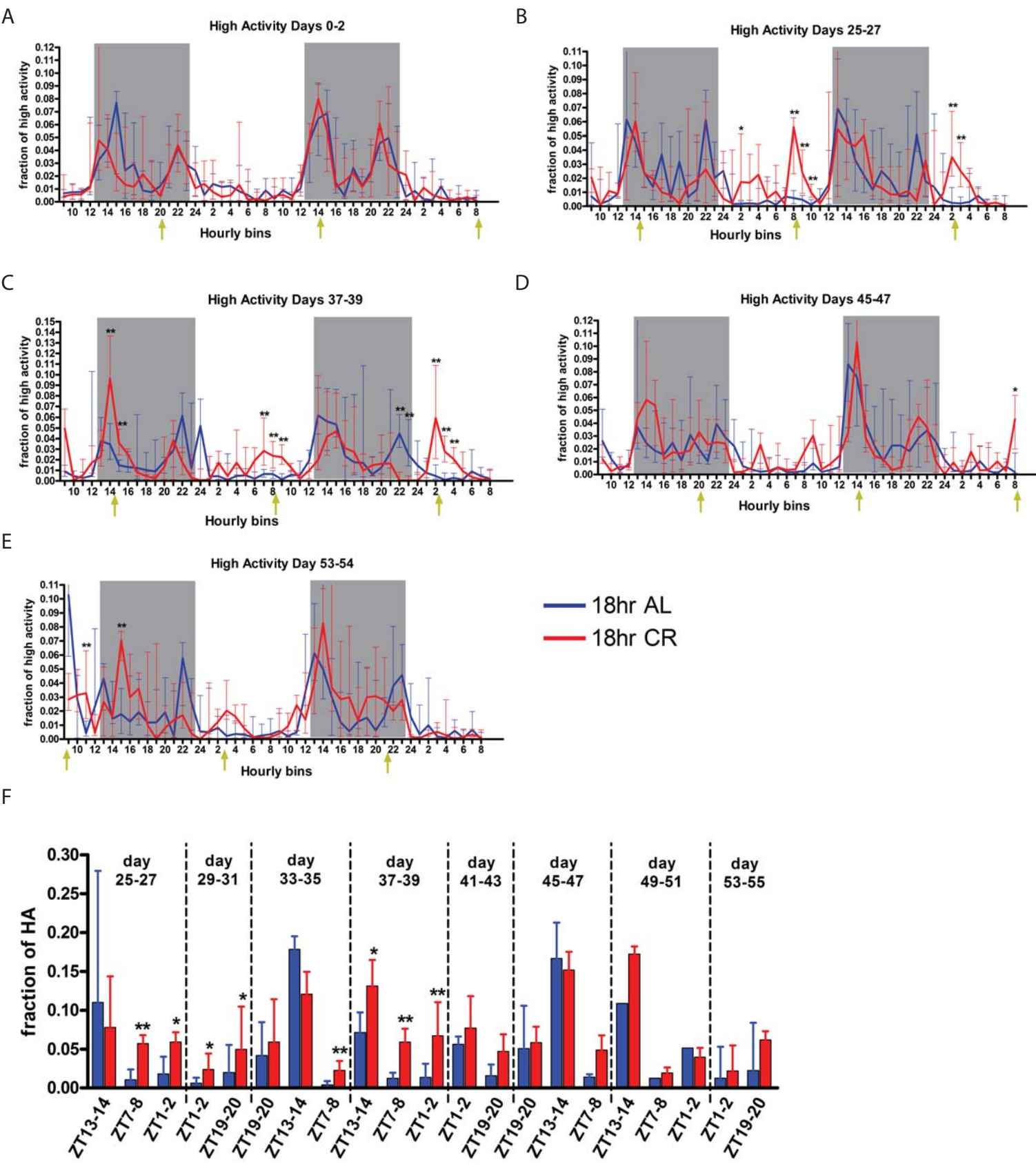

Supplement: Figure S7 — 18 hour interval feeding group median data shown for days 0–2, 25–27, 37–39, 45–47, and 53–54. The amount of normalized FAA during the 2 hours before each me is graphed in panel F. (PDF) [file pone.0037992.s007.pdf]

A

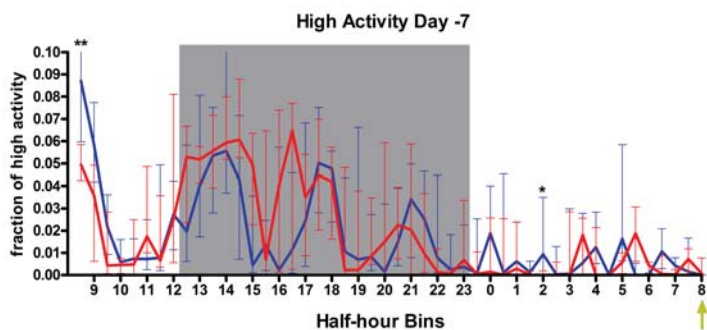

B

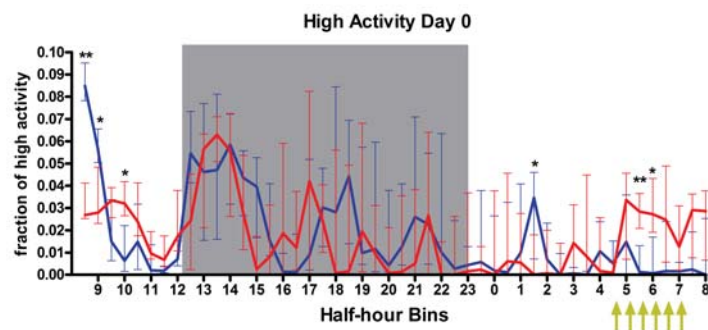

C

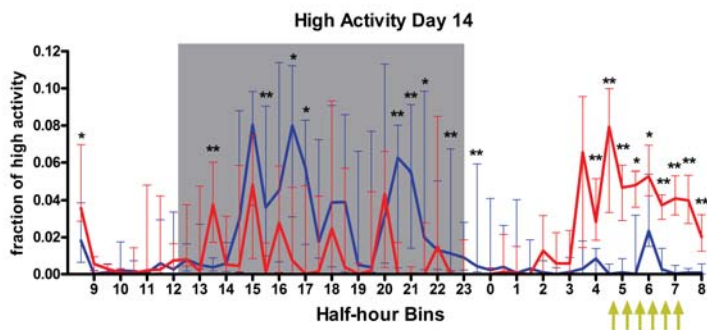

D

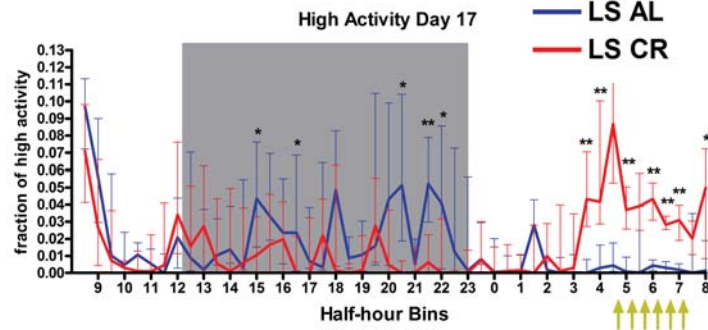

E

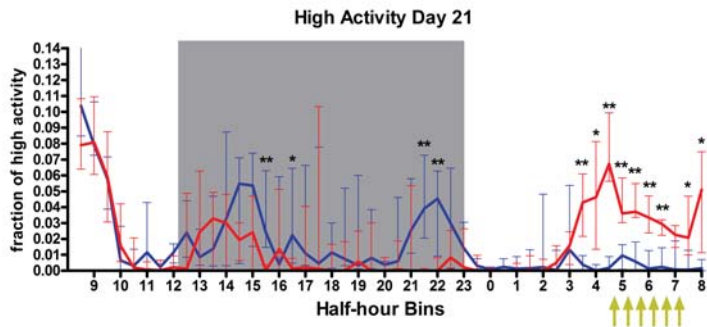

F

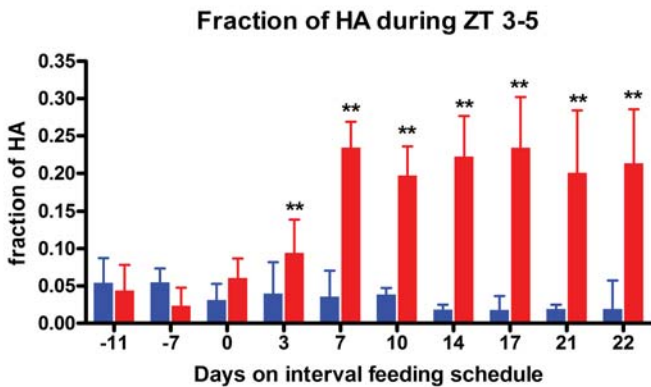

G

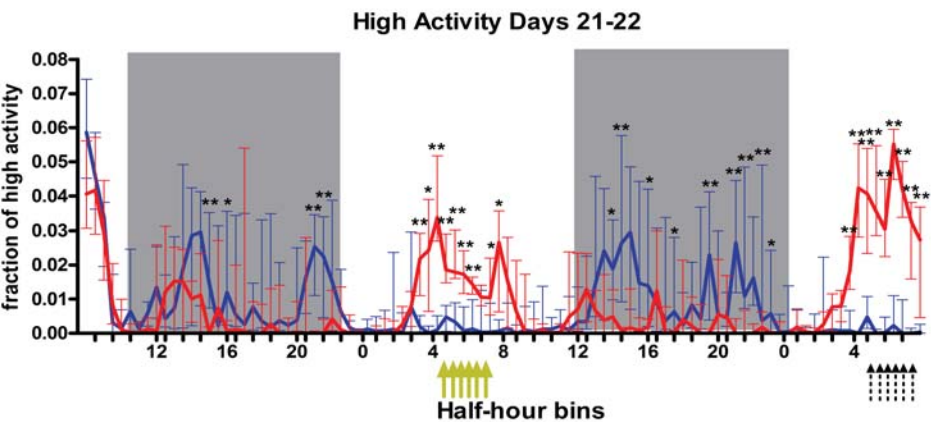

Supplement: Figure S9 — Short interval CR feeding experiment. (A) Fraction of high activity (in half hour bins) for AL and CR mice on Day −7. Day −7 is the first day of CR feeding, which occurred as a single feeding event at ZT 8. (B) Fraction of high activity for AL and CR mice on day 0, which was the first day of 30 minute interval feeding but the 7th day of 60% CR. The yellow arrows indicate the 6 feeding times (C) Day 14 of LS interval feeding (day 21 of CR). (D) Fraction of high activity for AL and CR mice on day 17 of LS interval feeding (day 24 of CR). (E) Fraction of high activity for AL and CR mice on day 21 of LS interval feeding (day 28 of CR). (F) Fraction of daily high activity for AL and CR mice during ZT 3–5 (2 h preceding feeding) at every recorded day of LS interval feeding. (G) Fraction of high activity for AL and CR mice on days 21 and 22 of LS interval feeding (days 28 and 29 of CR). n = 6 for both LS AL and LS CR at all time points. Yellow arrows represent feeding times and dotted black arrows indicate times at which feeding times would normally have occurred if the 30 minute cycle had continued. (PDF) [file pone.0037992.s009.pdf]

Supplemental Figure 10

A

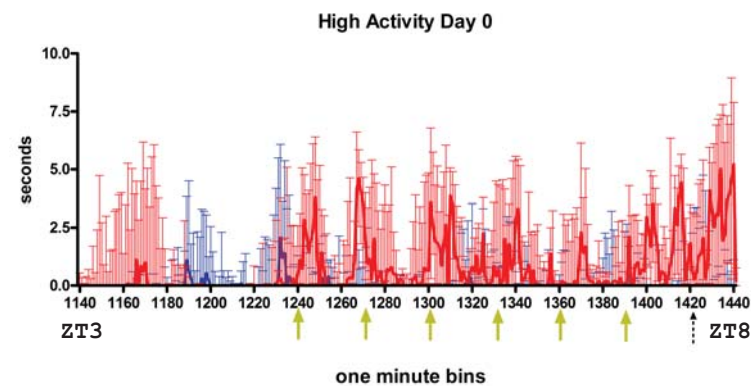

B

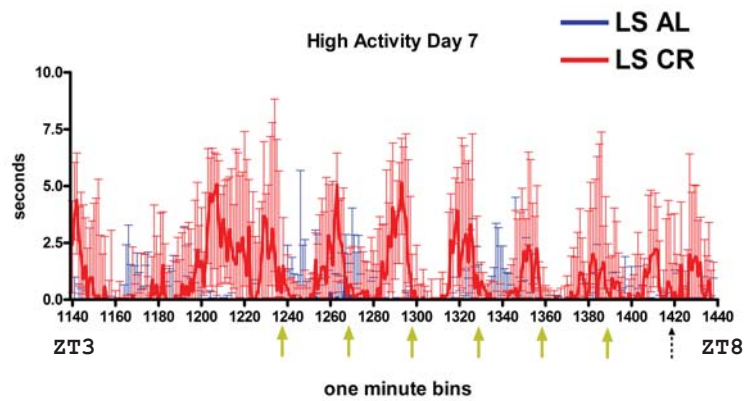

C

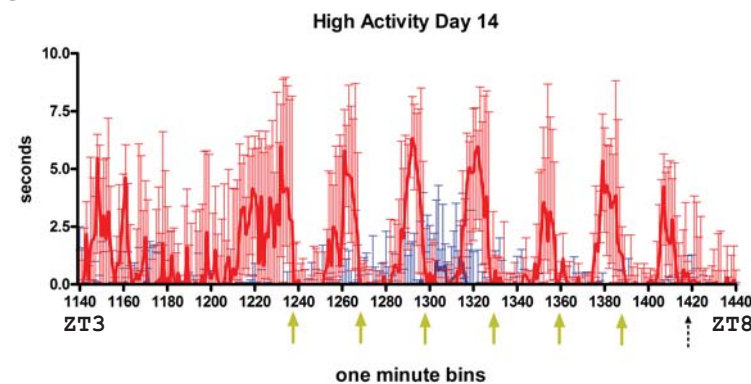

D

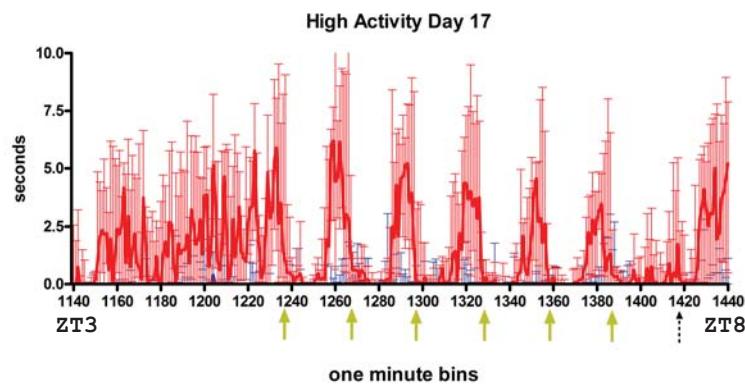

E

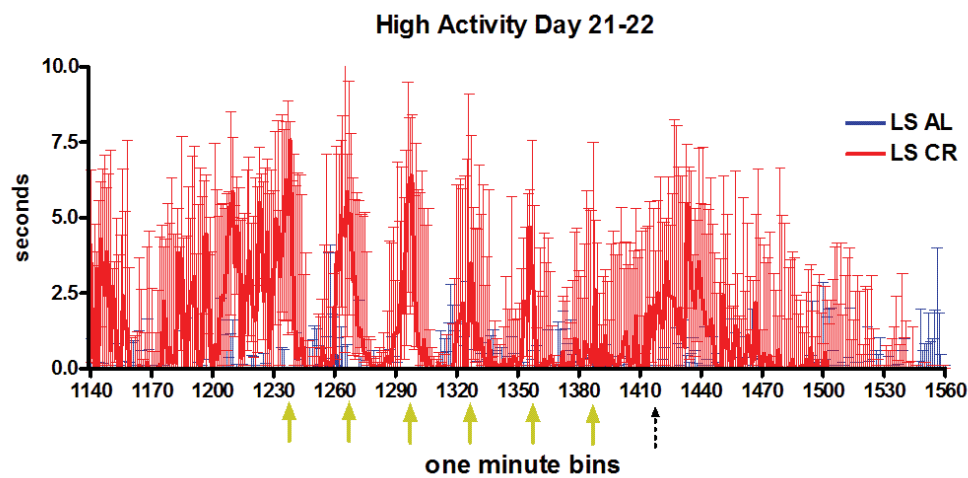

F

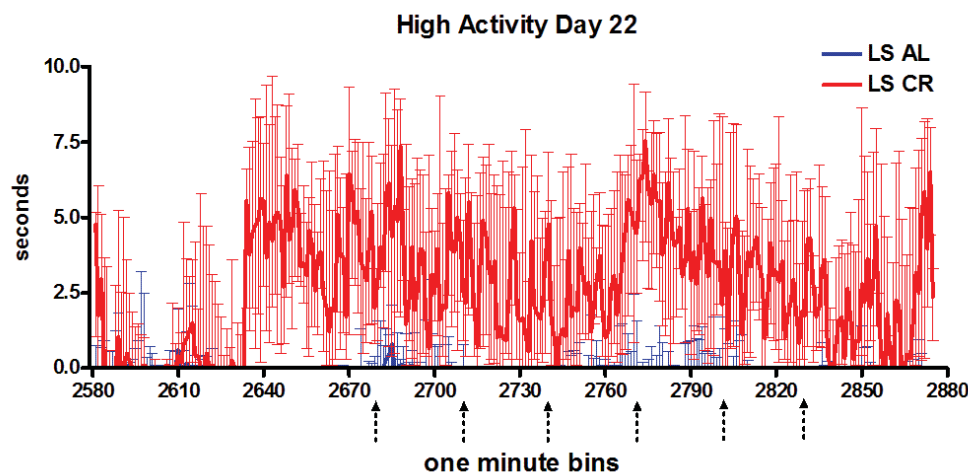

Supplement: Figure S10 — Anticipation of 30 minute meal delivery cycles. (A) High activity data (in seconds) for AL controls and LS CR mice is shown in 1 minute bins from ZT3-8 on Day 0 of LS interval feeding(the 7th day of CR). The x-axis is in minutes. Day 0 is the first day of LS interval feeding but day 7 of CR. (A) High activity data (in seconds) for AL controls and LS CR mice is shown in 1 minute bins from ZT3-8 on Day 0 of LS interval feeding (the 7th day of CR). Day 0 is the first day of LS interval feeding but day 7 of CR. (B) Seconds of high activity for AL and CR mice from ZT3-8 on day 7 of LS interval feeding (day 14 of CR). (C) Seconds of high activity for AL and CR mice from ZT3-8 on day 14 of LS interval feeding (day 21 of CR). (D) Seconds of high activity for AL and CR mice from ZT3-8 on day 17 of LS interval feeding (day 24 of CR). (E) Seconds of high activity data for day 21 and part of day 22. (F) Seconds of high activity for day 22 during the time at which meals were normally scheduled, but were withheld during this experiment. Normal feeding times indicated by dashed arrows. (PDF) [file pone.0037992.s010.pdf]

# Supplemental Figure 11

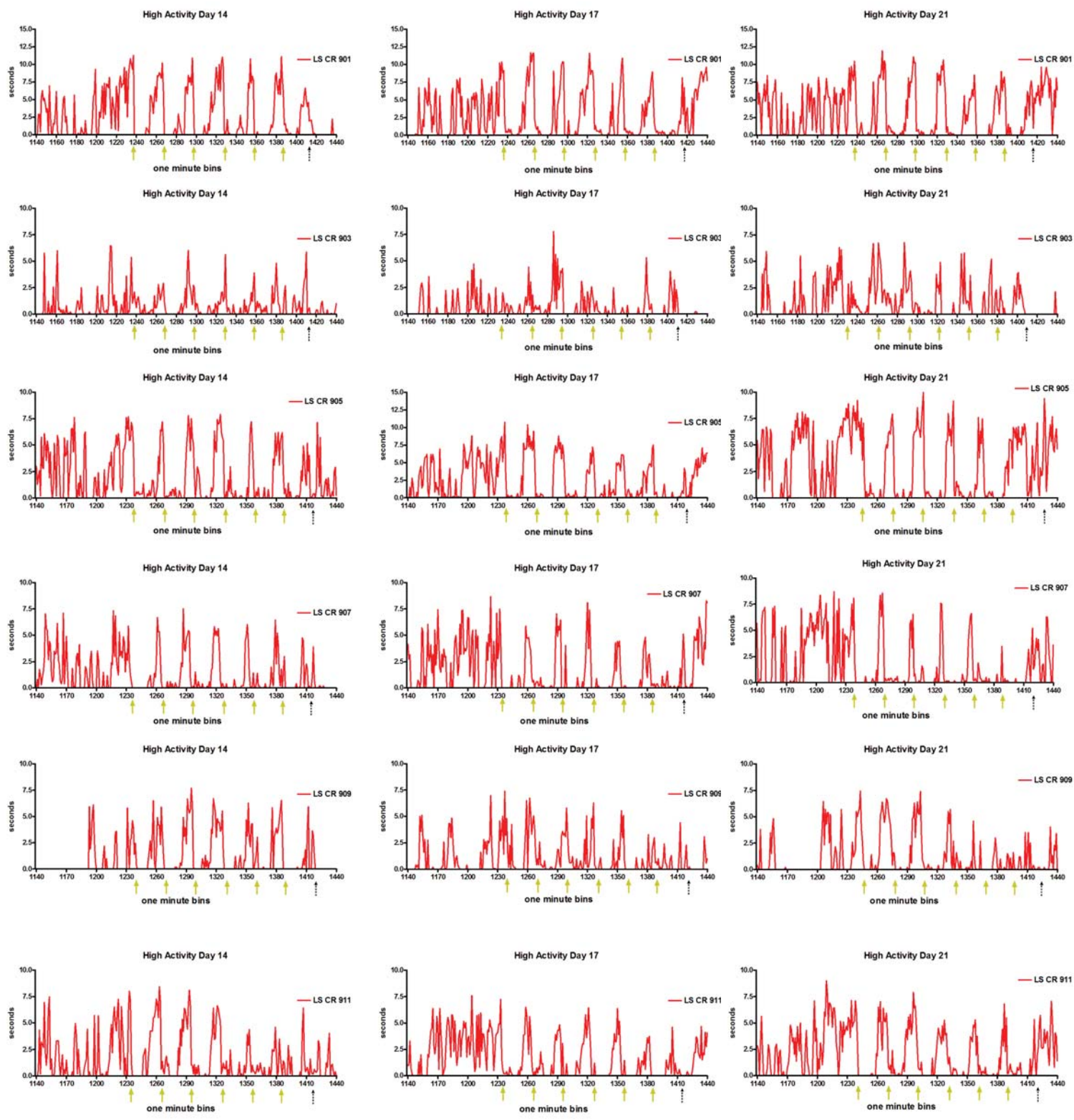

Supplement: Figure S11 — Individual LS CR mice high activity data in 1 min bins for days 14, 17, and 21 shown for mouse 901, 903, 905, 907, 909, and 911. (PDF) [file pone.0037992.s011.pdf]

Supplemental Figure 12

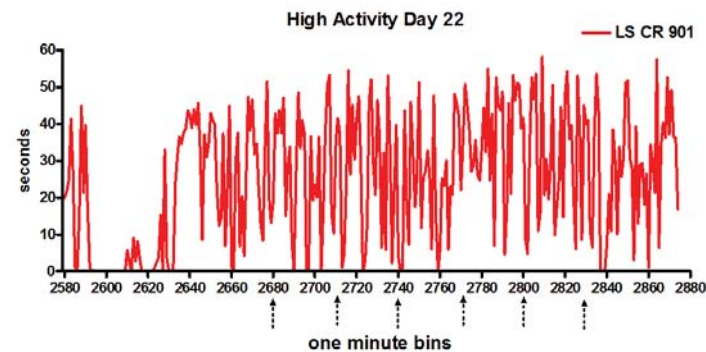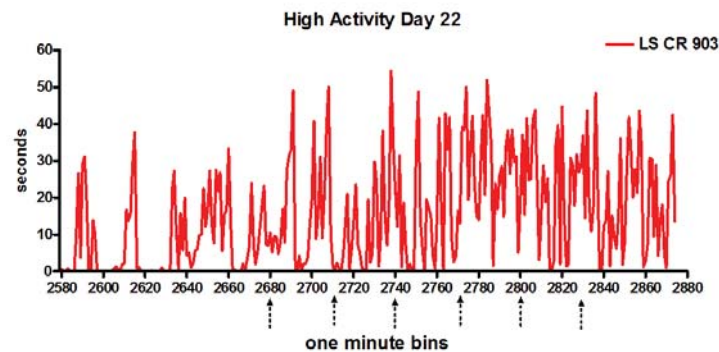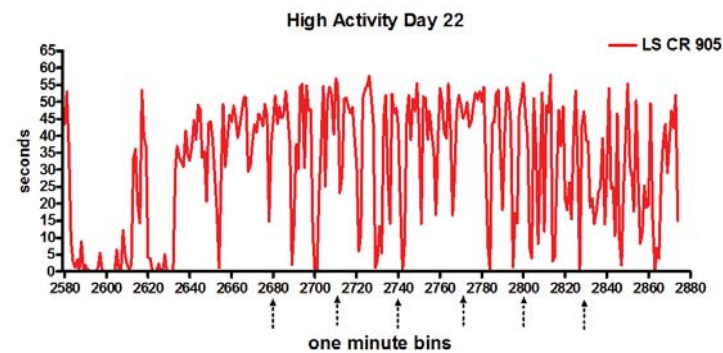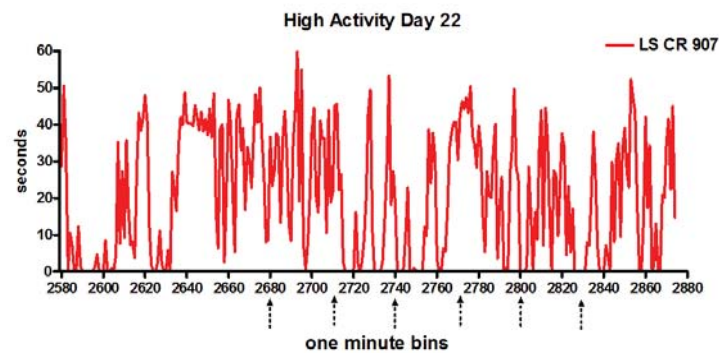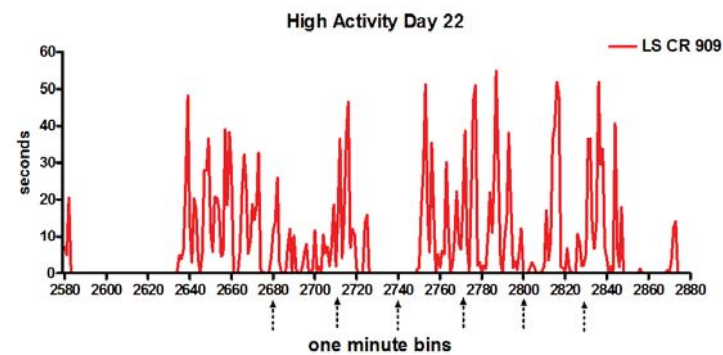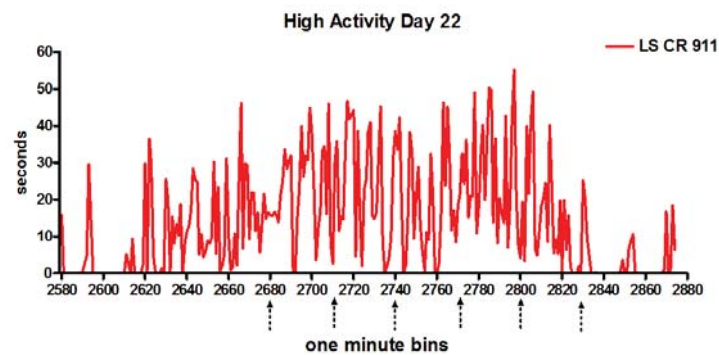

Supplement: Figure S12 — Short interval feeding schedule. Seconds of high activity for LS CR mice 901, 903, 905, 907, 909, and 911 for day 22 when meals were not delivered. The 30 minute intervals when food was normally delivered are indicated by black arrows. Minute 1140 corresponds to ZT 3 and minute 1140 corresponds to ZT 8. (PDF) [file pone.0037992.s012.pdf]

**Meal Duration (10% CR/meal)**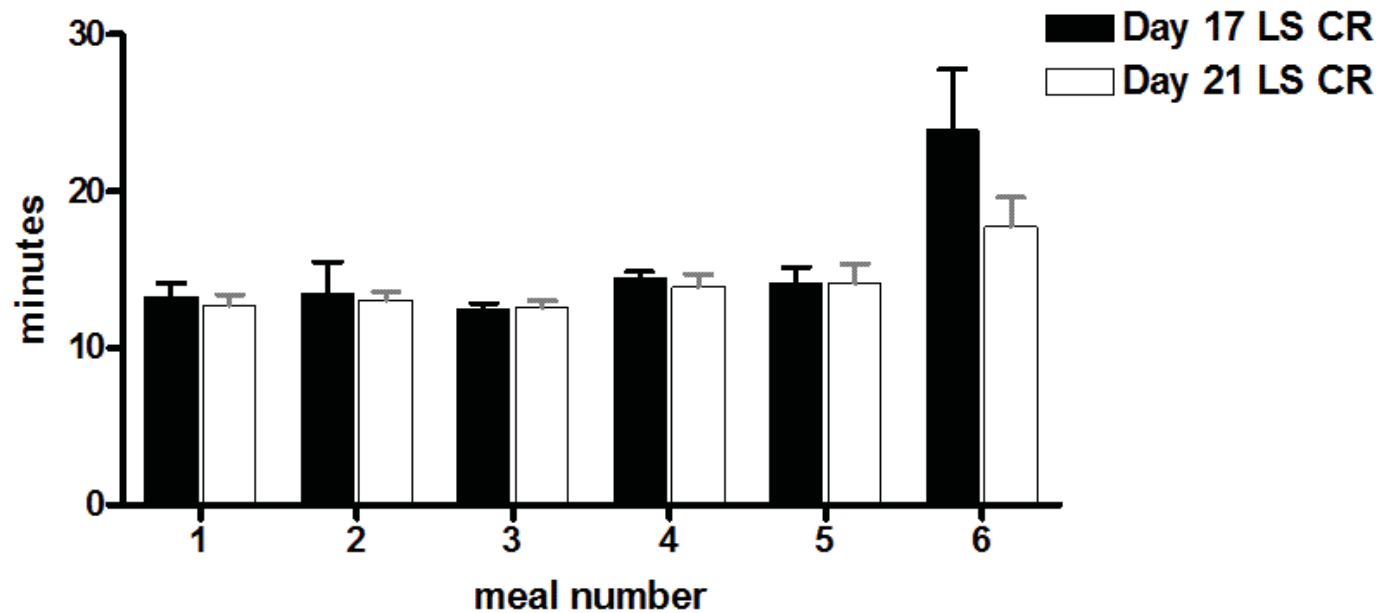

Supplement: Figure S13 — Quantification of meal duration. Meal duration was quantified by manually scoring the amount of time each mouse spent eating after food delivery for day 17 and day 21 of LS interval feeding. For day 17, n = 4 mice and for day 21, n = 6 mice. (PDF) [file pone.0037992.s013.pdf]
