# Supplementary material for: Food Anticipatory Activity Behavior of Mice across a Wide Range of Circadian and Non-Circadian Intervals
Source: PLoS One. 2012 May 25;7(5):e37992. doi: 10.1371/journal.pone.0037992 (PMC3360658; doi:10.1371/journal.pone.0037992)

Supplemental Figure 4

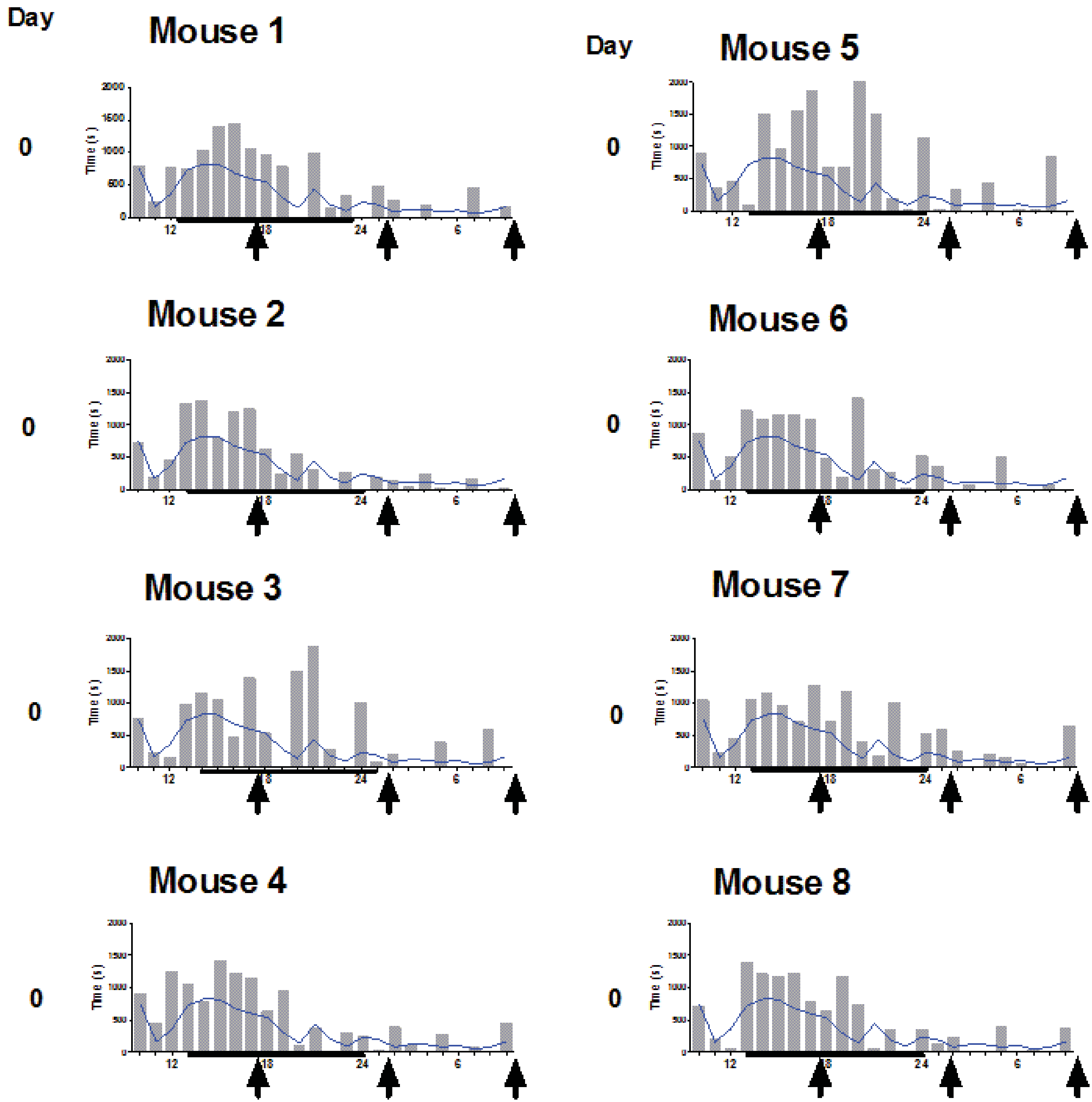

Supplemental Figure 4 (continued)

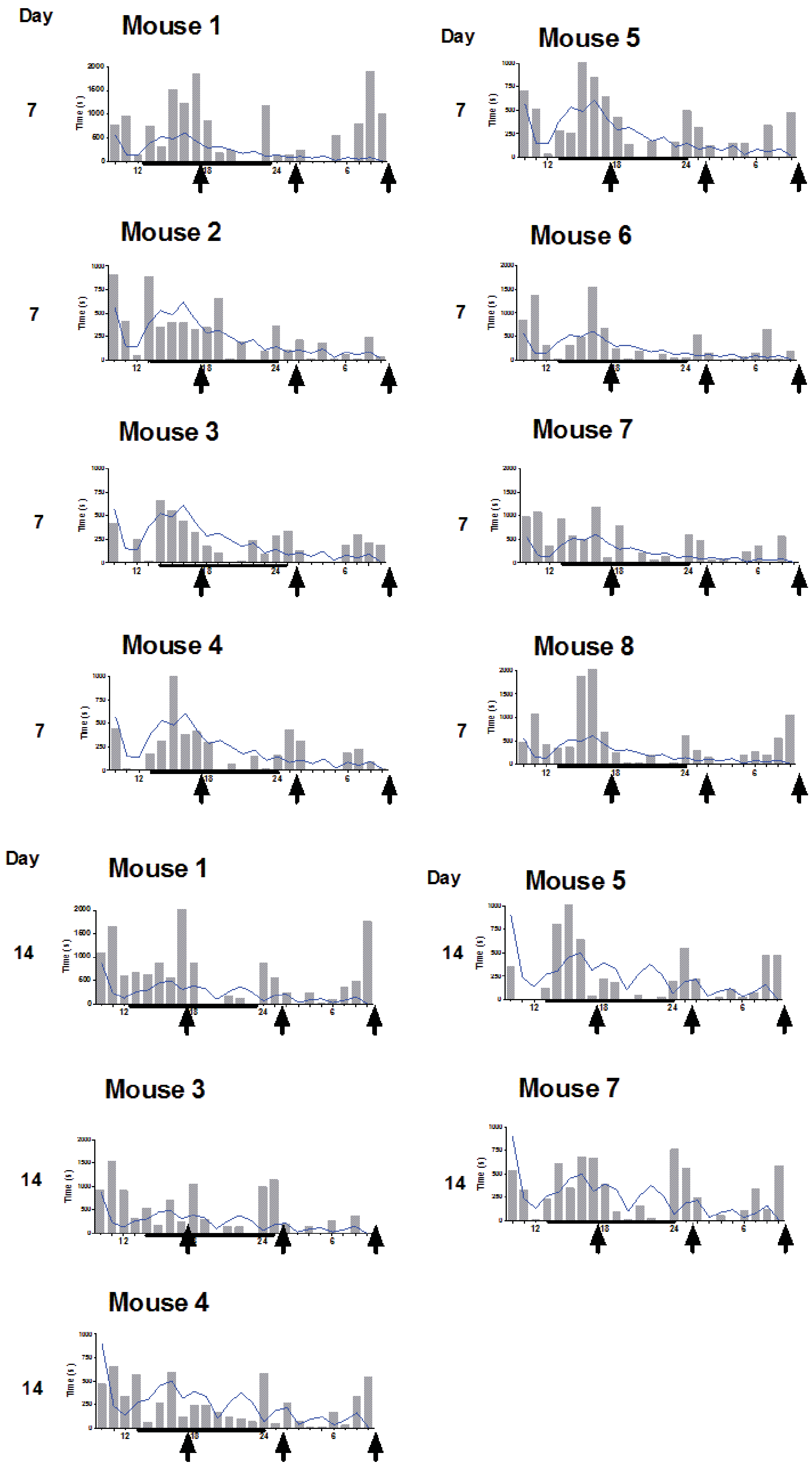

Supplemental Figure 4 (continued)

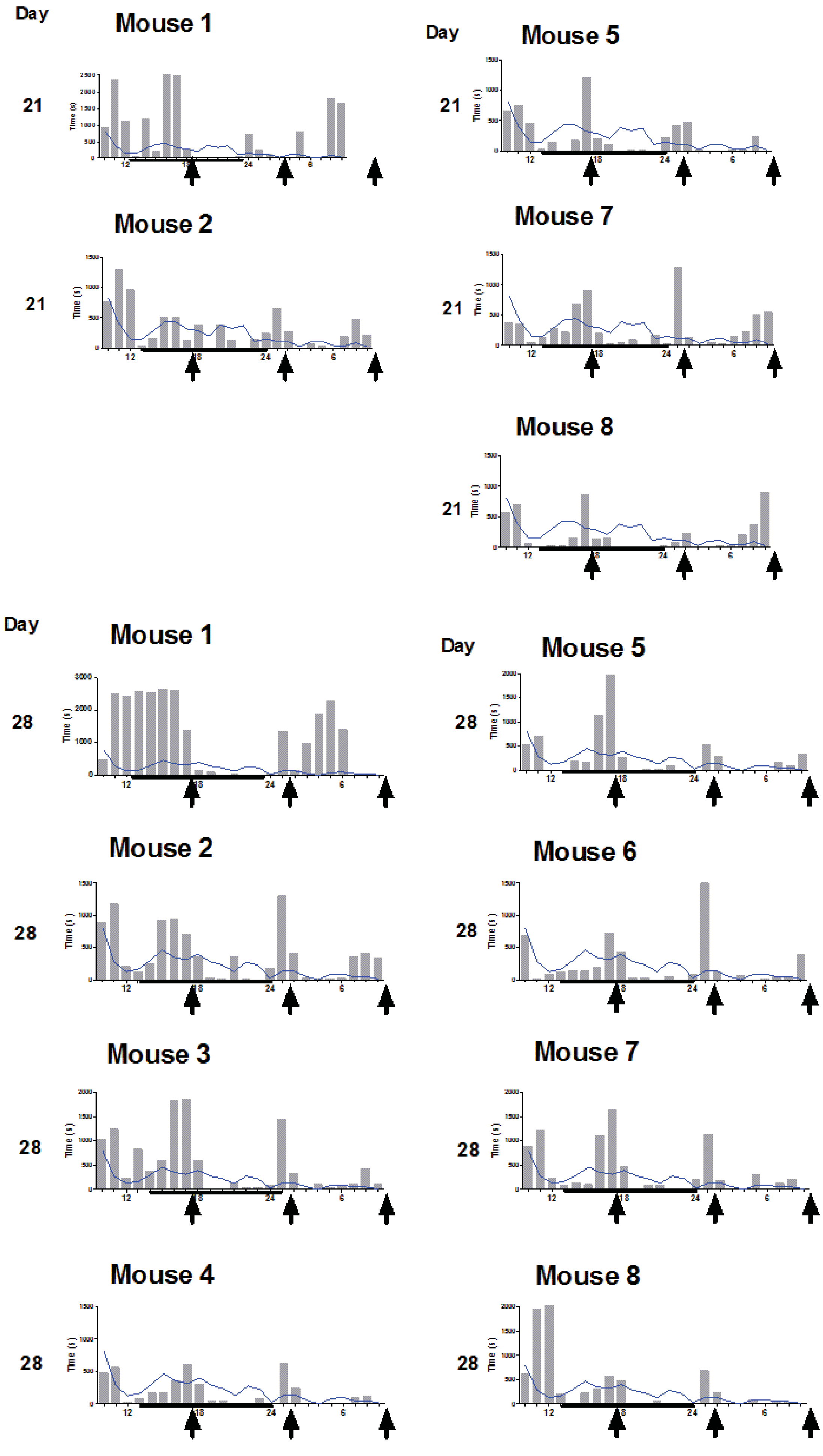

Supplemental Figure 4 (continued)

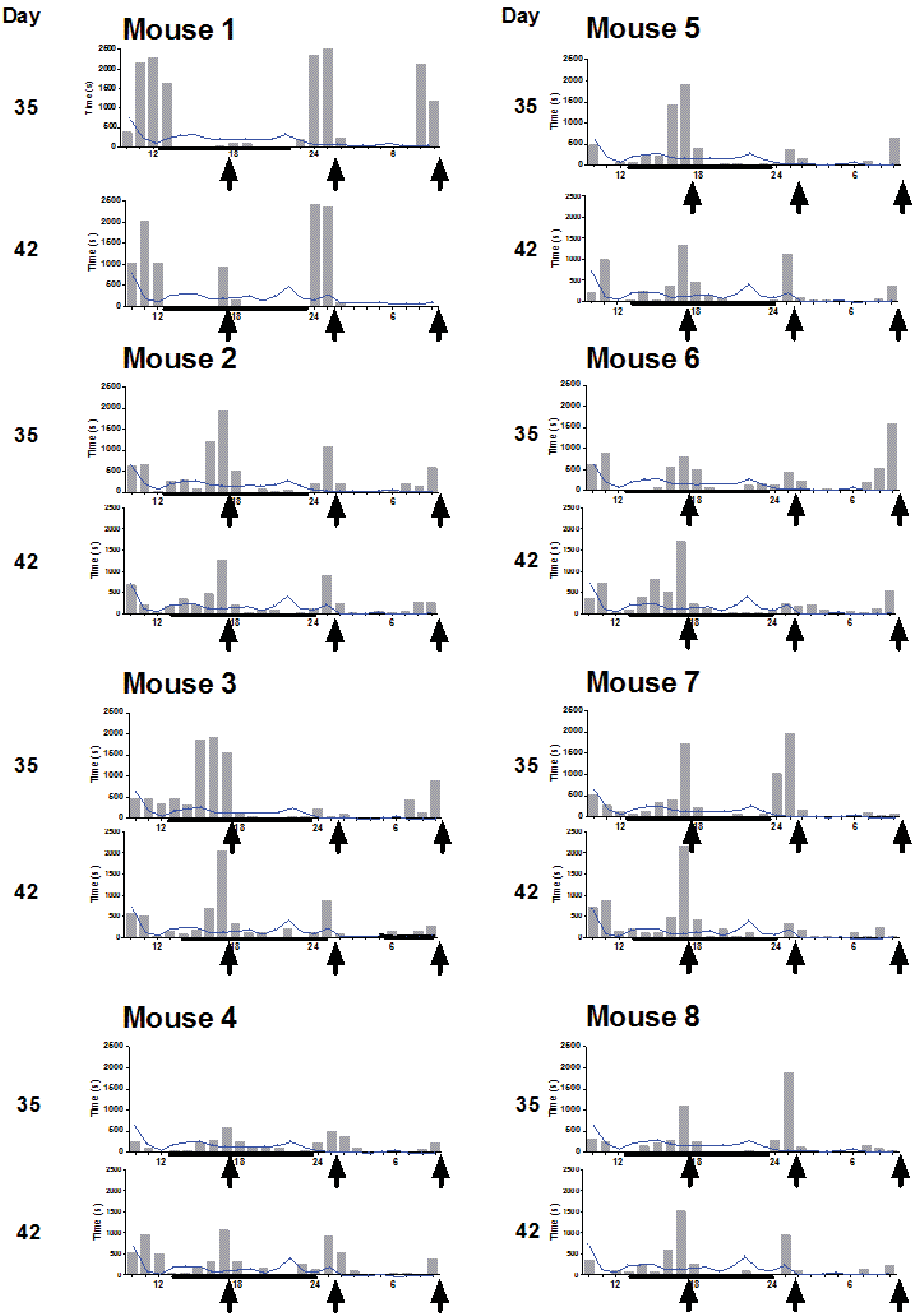

Supplement: Figure S4 — Individual mouse data for 8 hour interval feeding schedule for days 0, 7, 14, 21, 28, 35, and 42. Blue line indicates mean AL control activity. (PDF) [file pone.0037992.s004.pdf]
