# Supplementary material for: Food Anticipatory Activity Behavior of Mice across a Wide Range of Circadian and Non-Circadian Intervals
Source: PLoS One. 2012 May 25;7(5):e37992. doi: 10.1371/journal.pone.0037992 (PMC3360658; doi:10.1371/journal.pone.0037992)

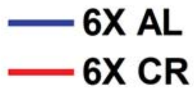

Supplemental Figure 6

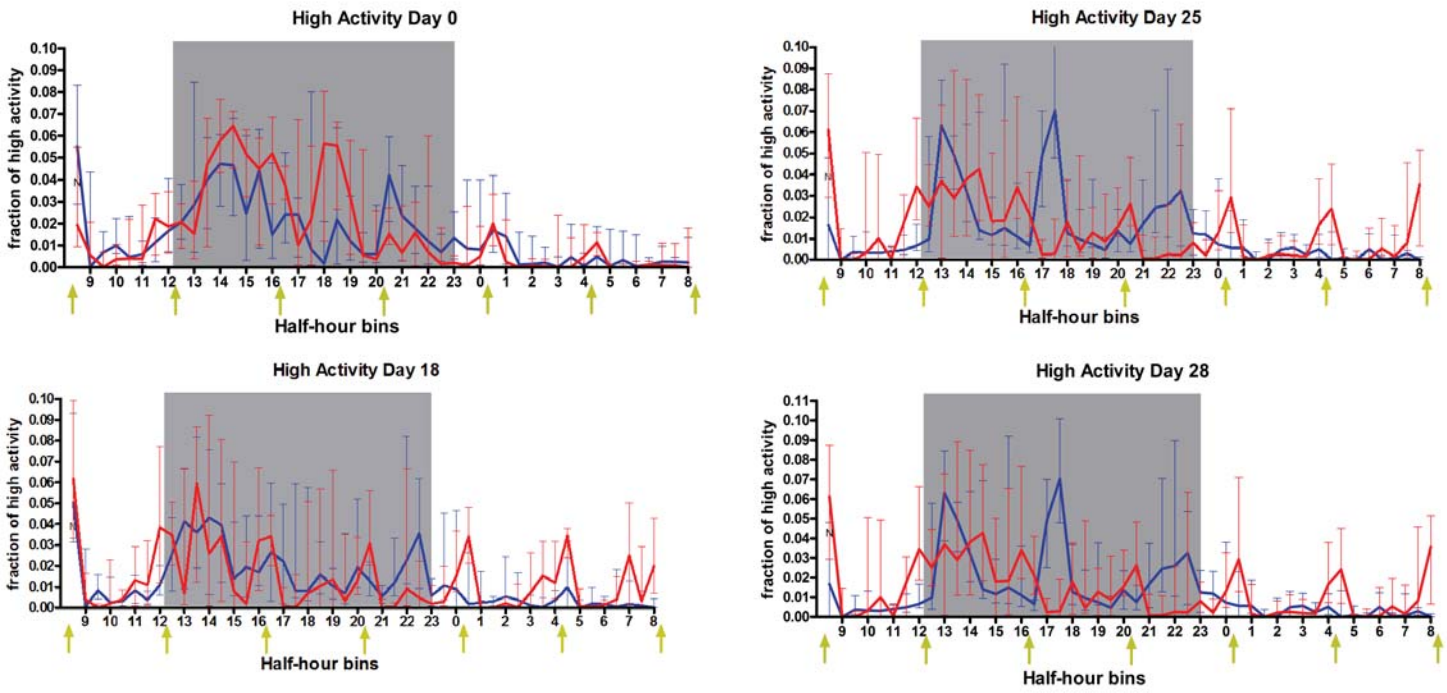

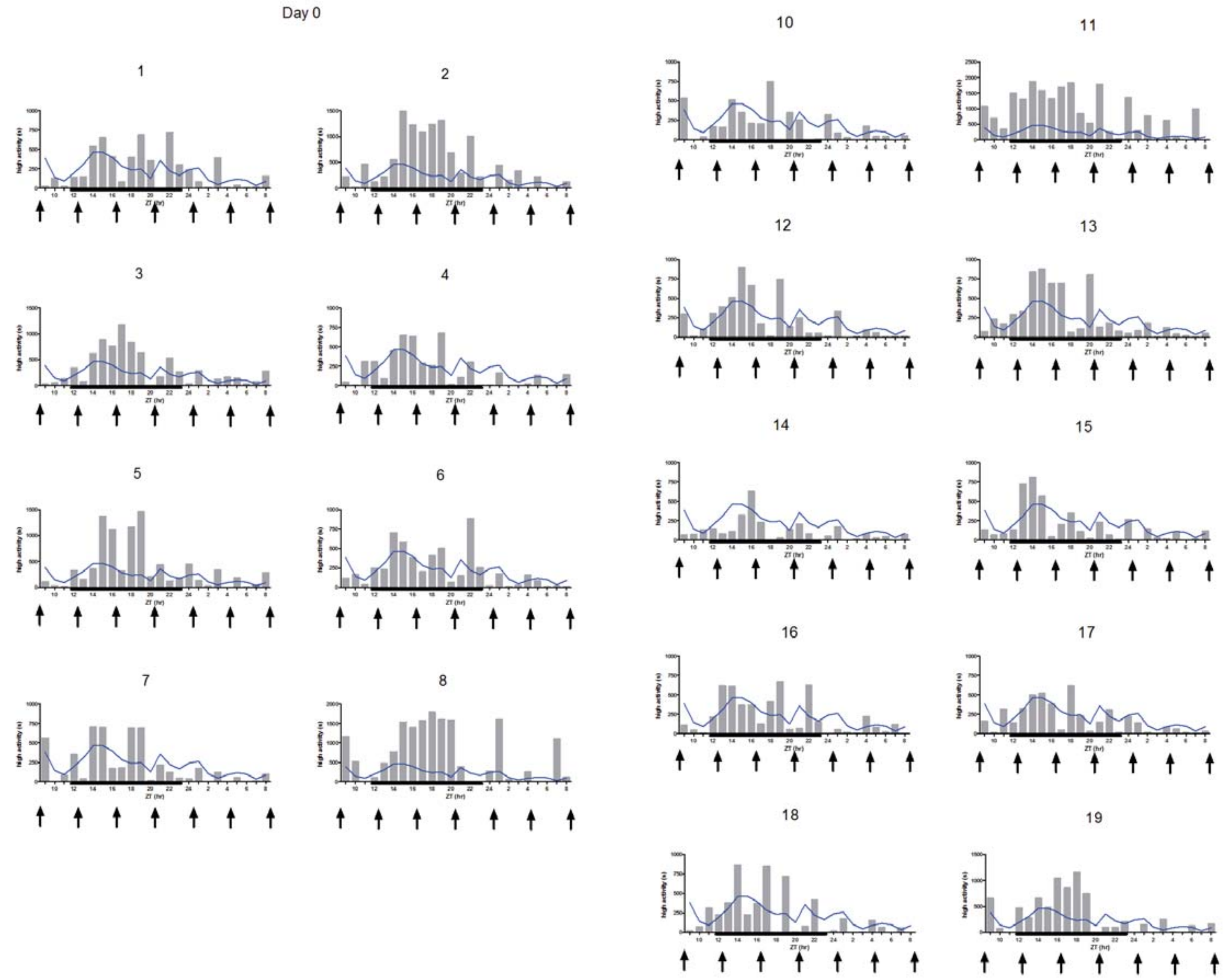

Supplemental Figure 6 (continued)

Day 10

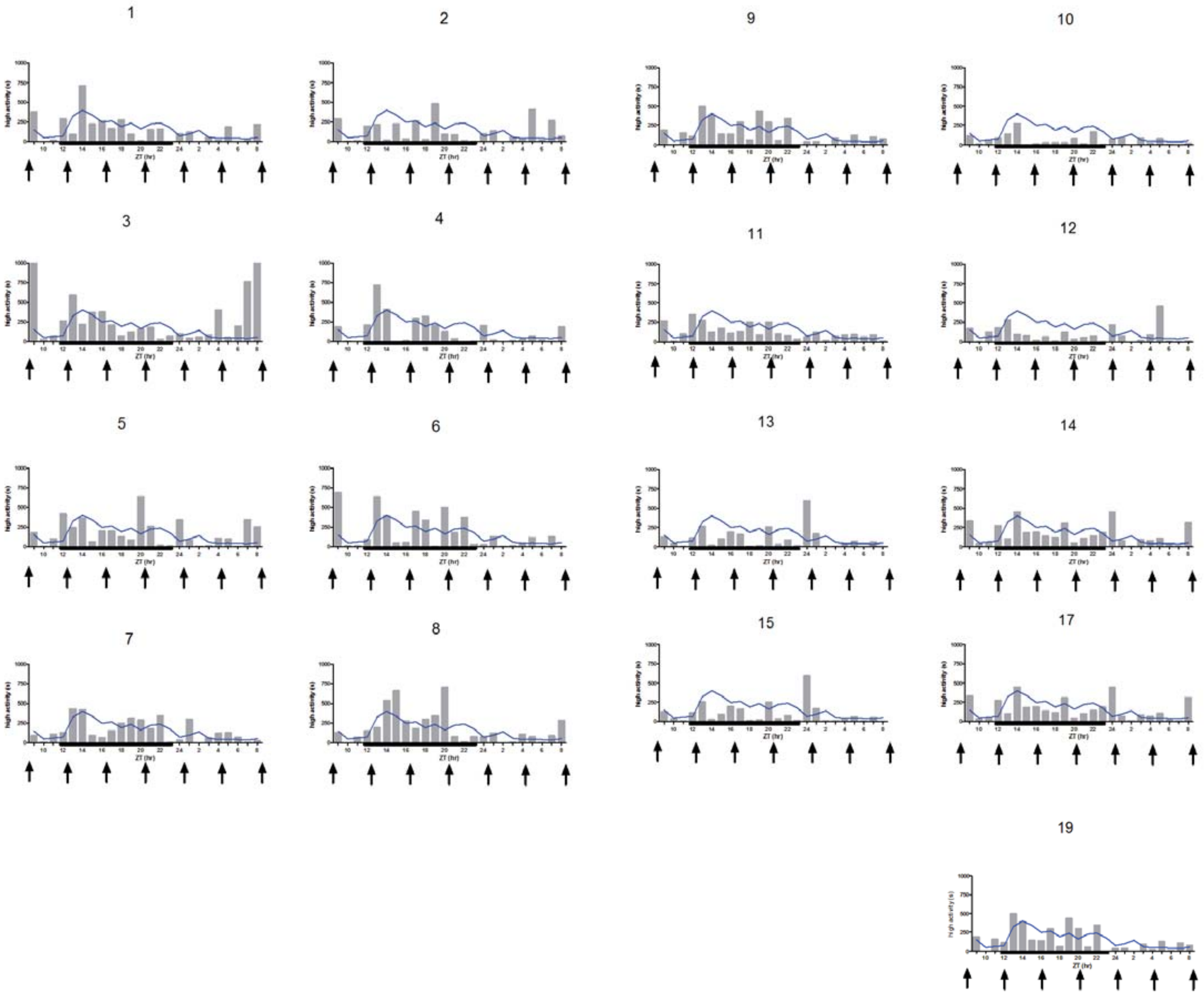

Supplemental Figure 6 (continued)

Day 14

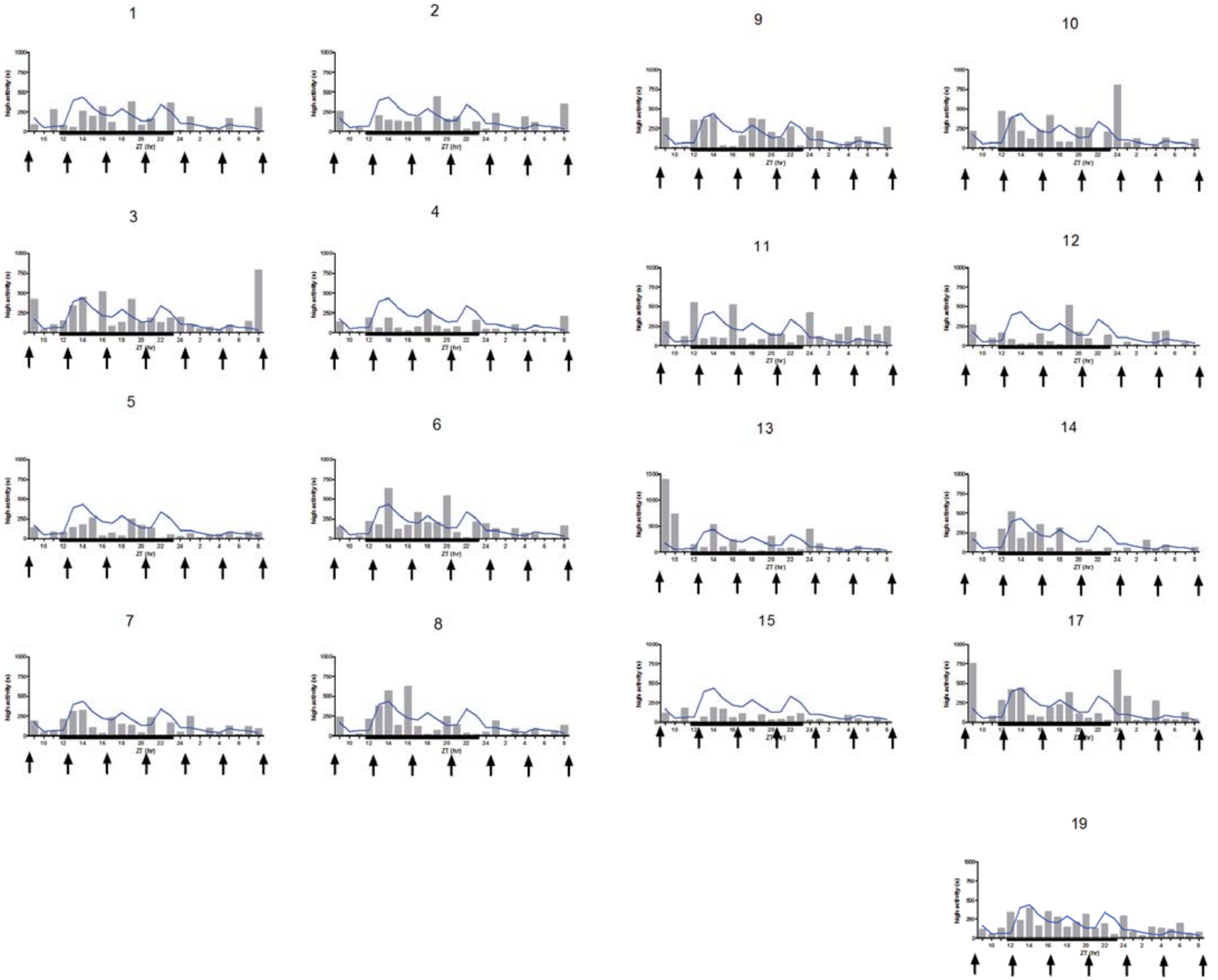

Supplemental Figure 6 (continued)

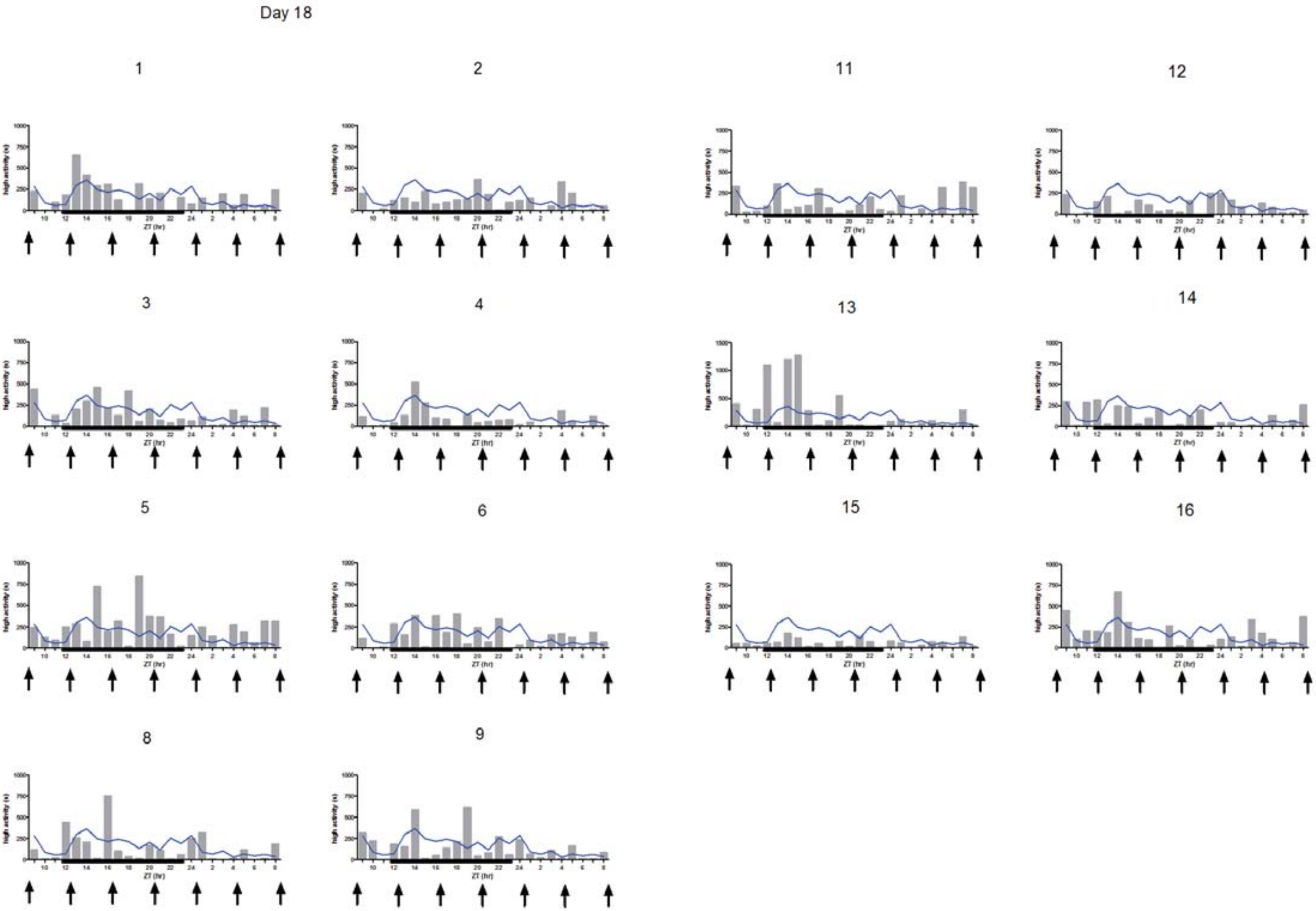

Supplemental Figure 6 (continued)

Day 21

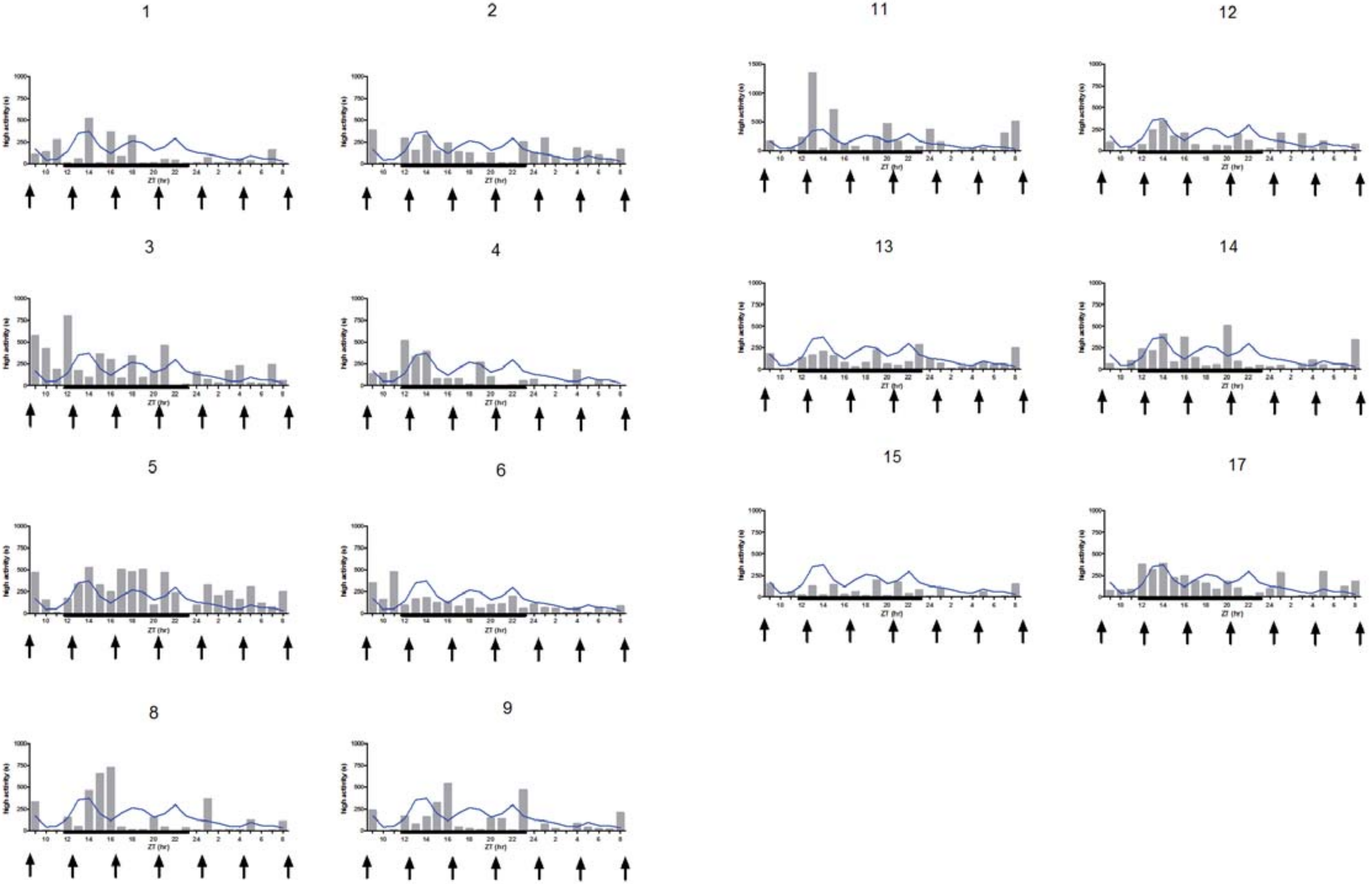

Supplemental Figure 6 (continued)

Day 24

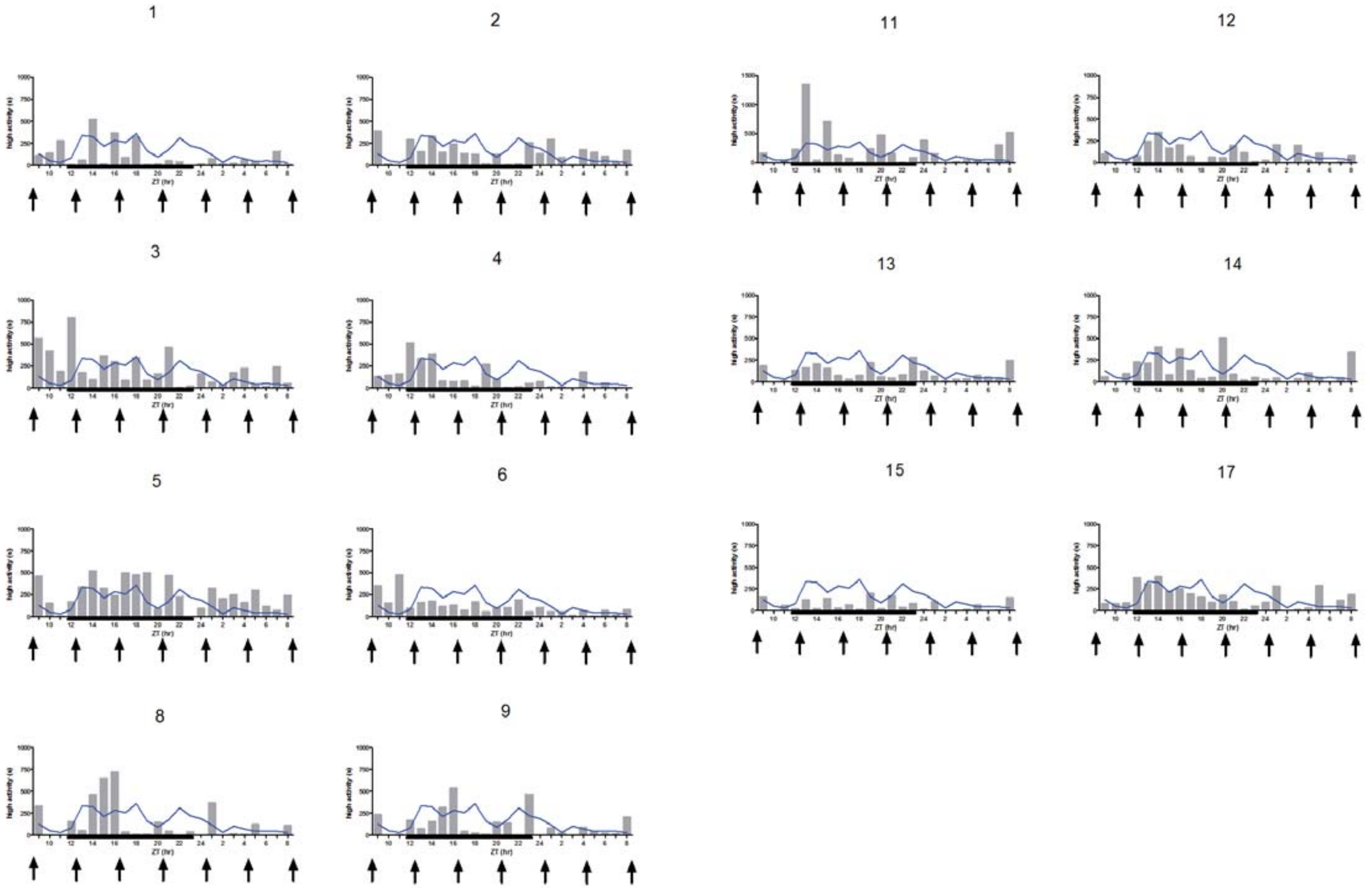

Supplemental Figure 6 (continued)

Day 29

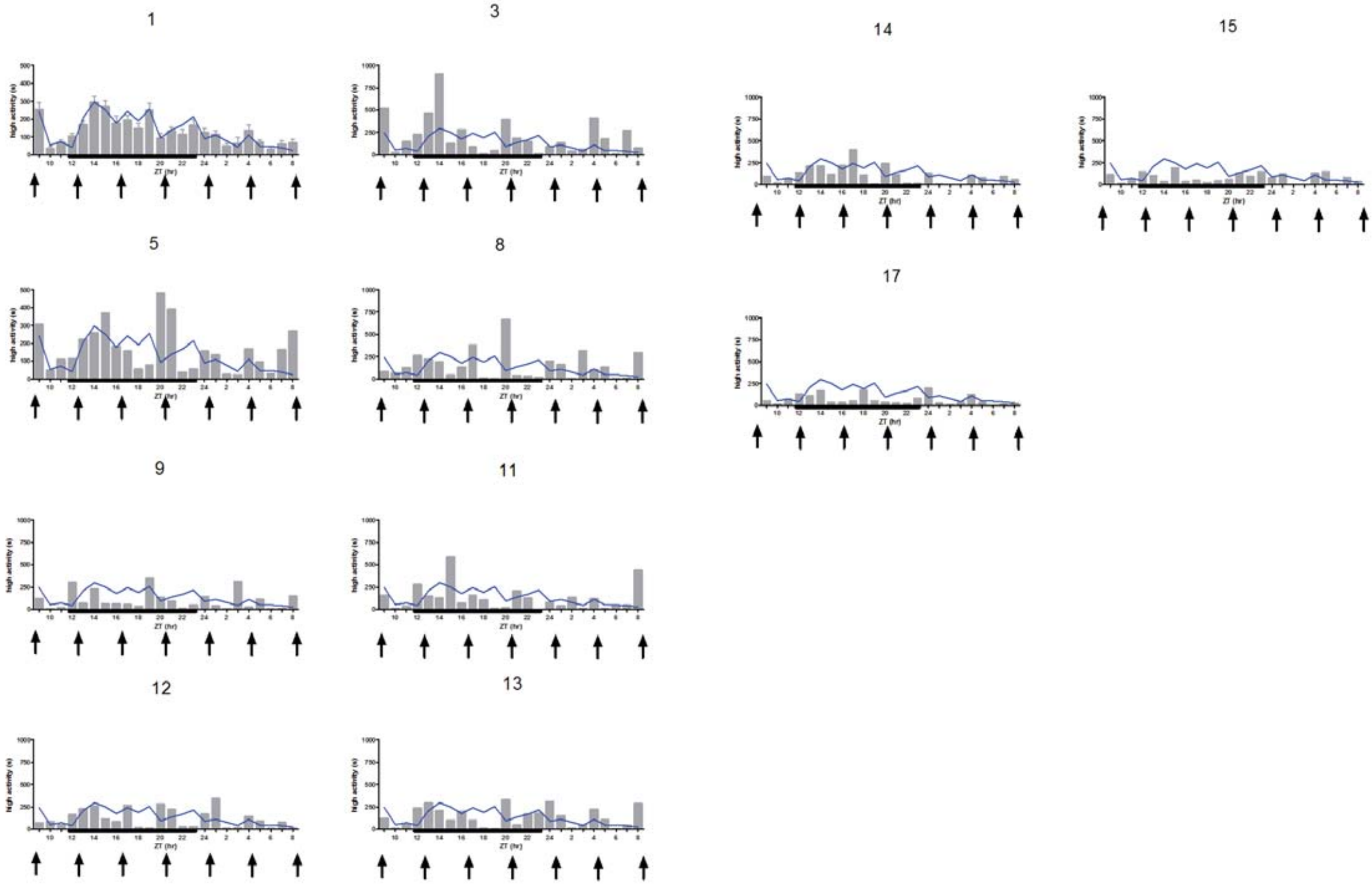

Day 32

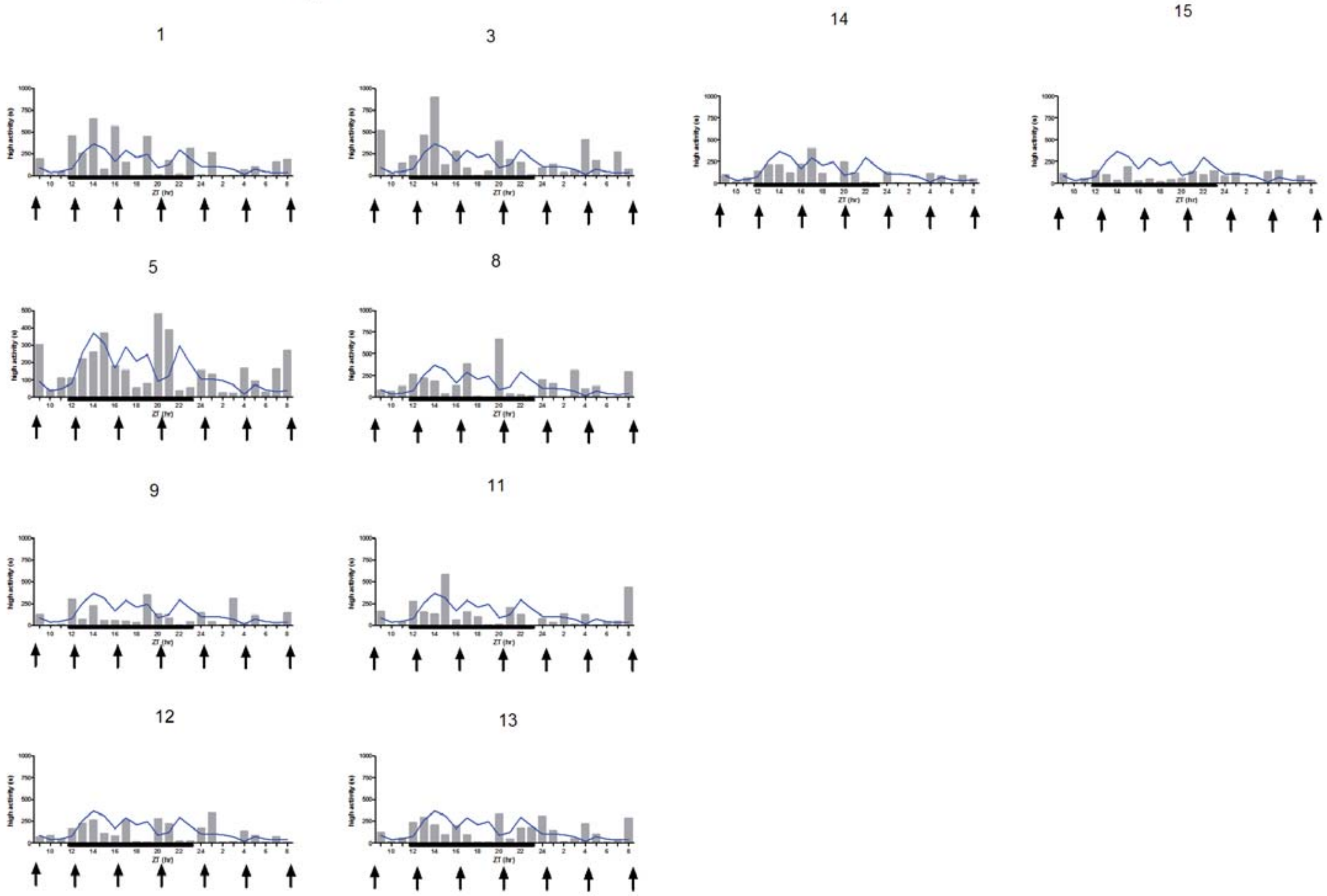

Supplemental Figure 6 (continued)

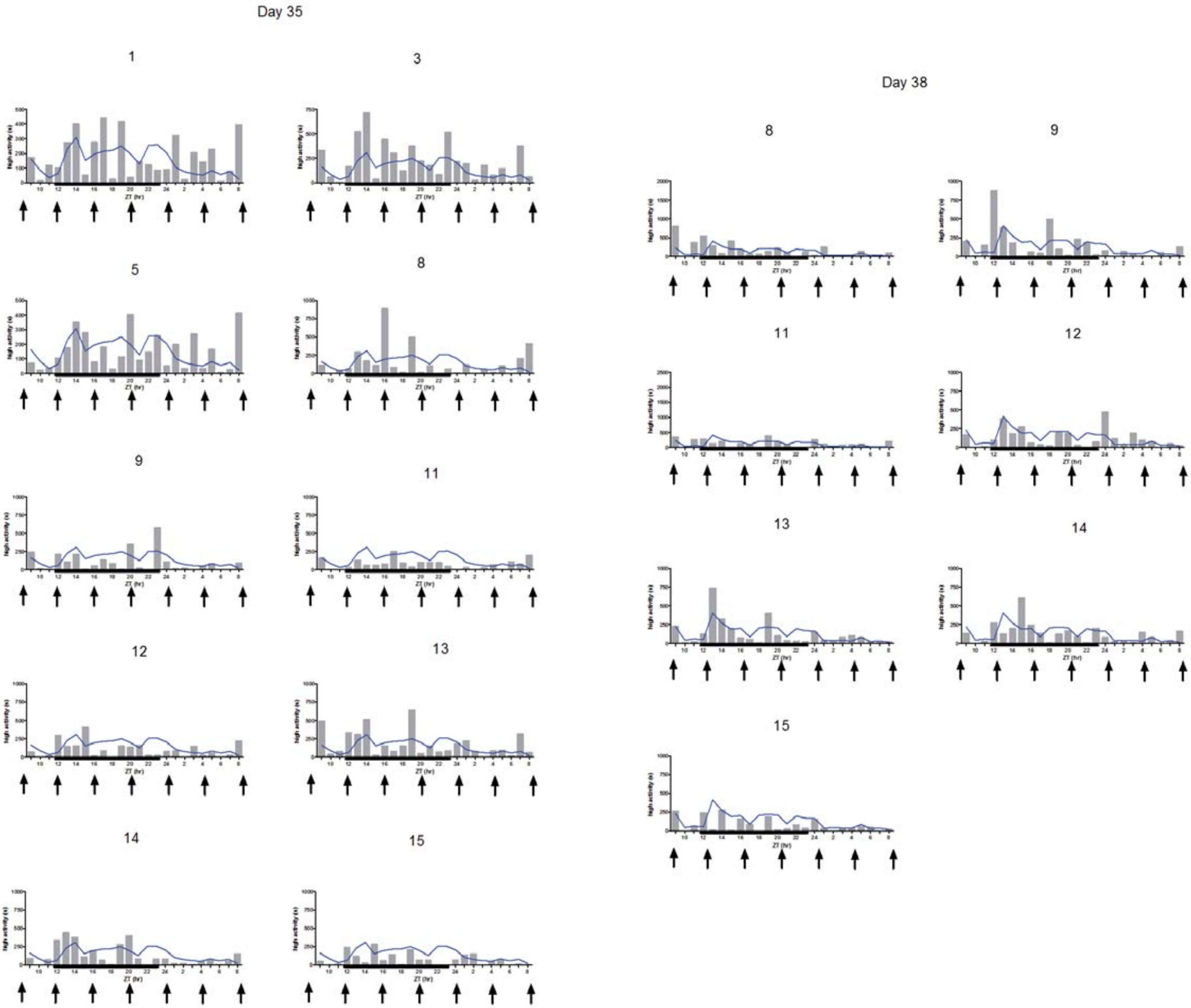

Supplemental Figure 6 (continued)

Days 42-43

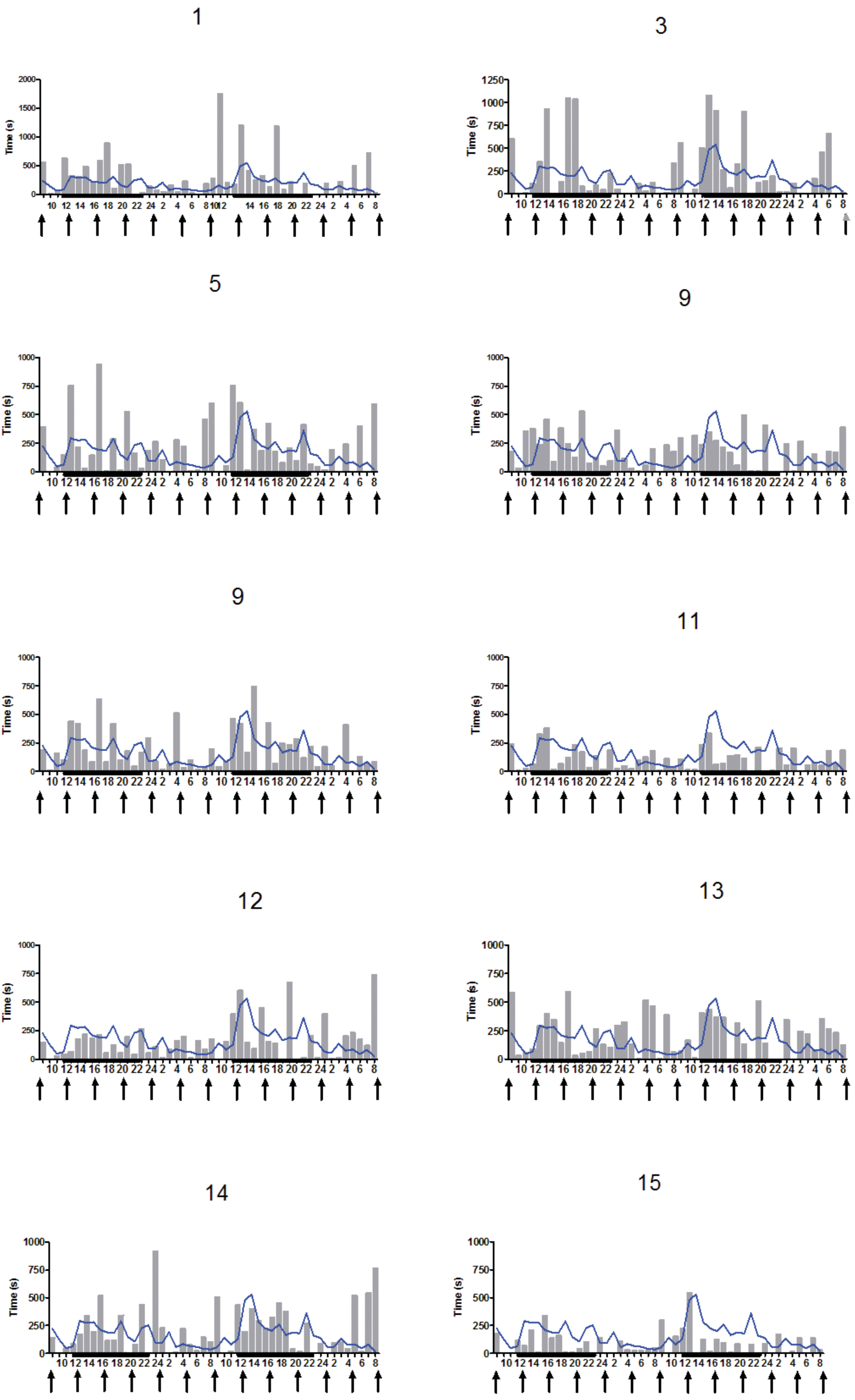

Supplement: Figure S6 — 4 hour interval CR feeding group data for days 0 and 28. Individual mouse data from 4 hour interval fed CR mice on days 0, 10, 18, 21, 28, 32, 35, 38 and 42–43. (PDF) [file pone.0037992.s006.pdf]
