# Supplementary material for: Food Anticipatory Activity Behavior of Mice across a Wide Range of Circadian and Non-Circadian Intervals
Source: PLoS One. 2012 May 25;7(5):e37992. doi: 10.1371/journal.pone.0037992 (PMC3360658; doi:10.1371/journal.pone.0037992)

Supplemental Figure 8

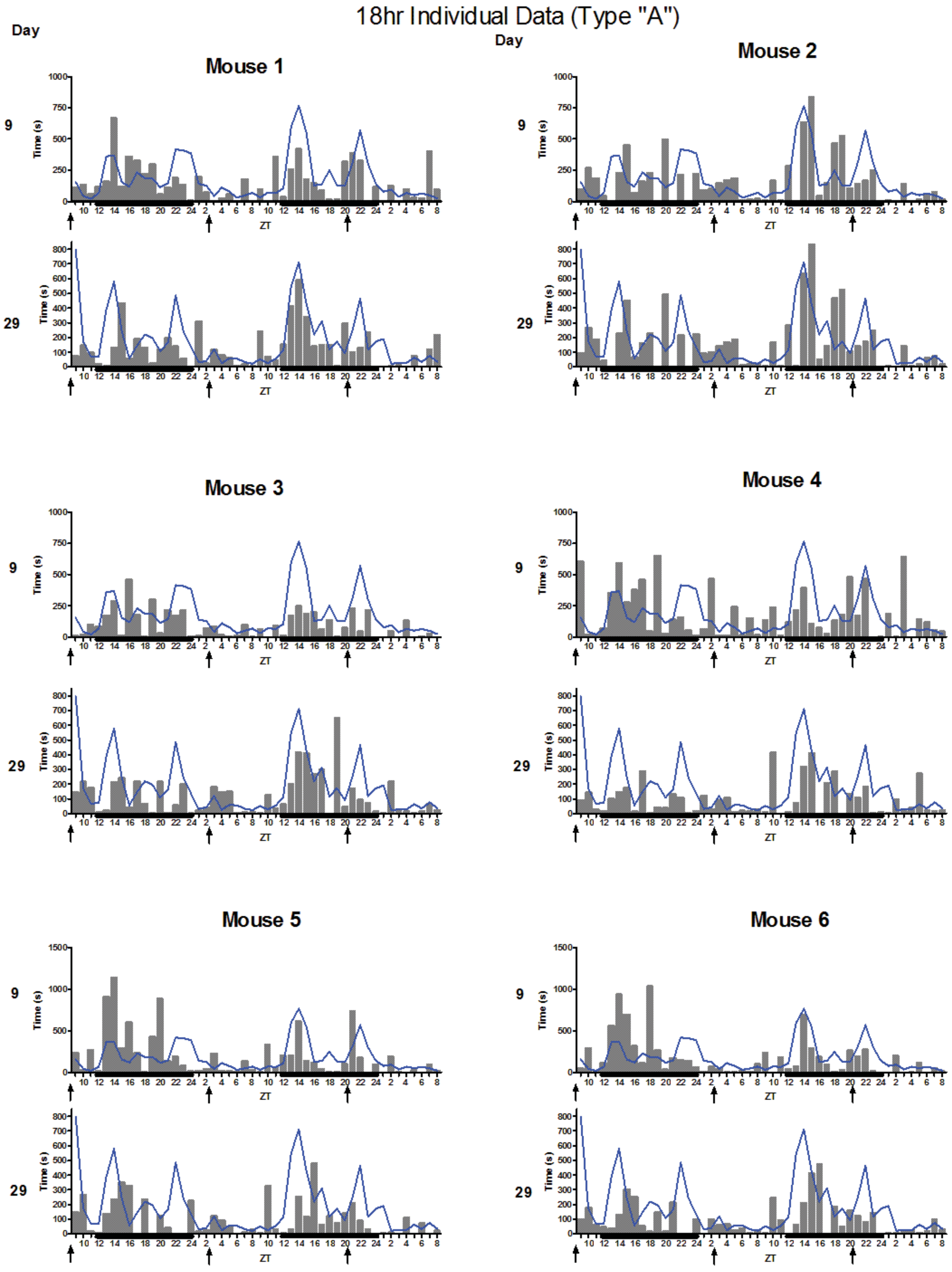

Supplemental Figure 8 (continued)

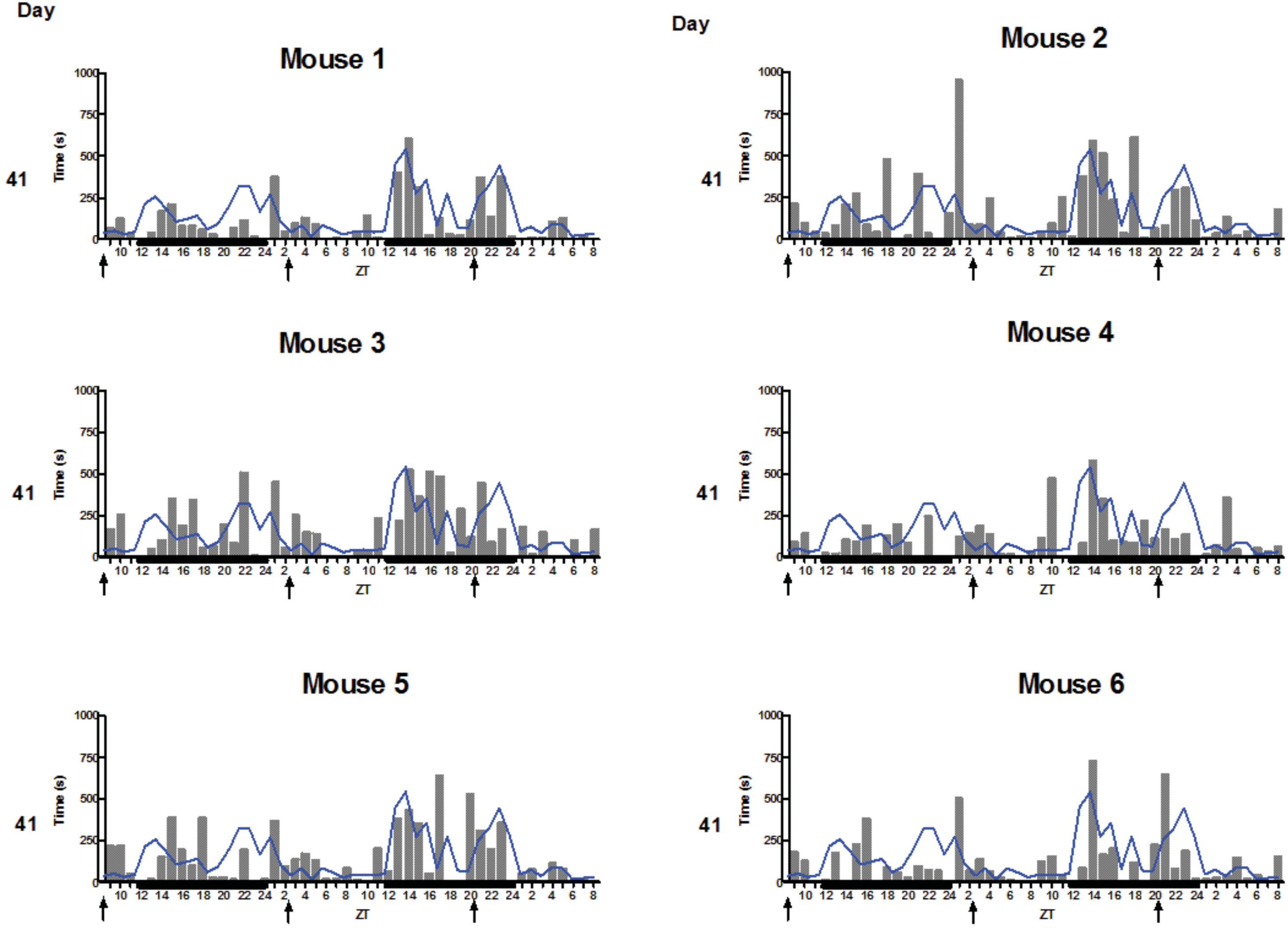

Supplemental Figure 8 (continued)

18hr Individual Data (Type "B")

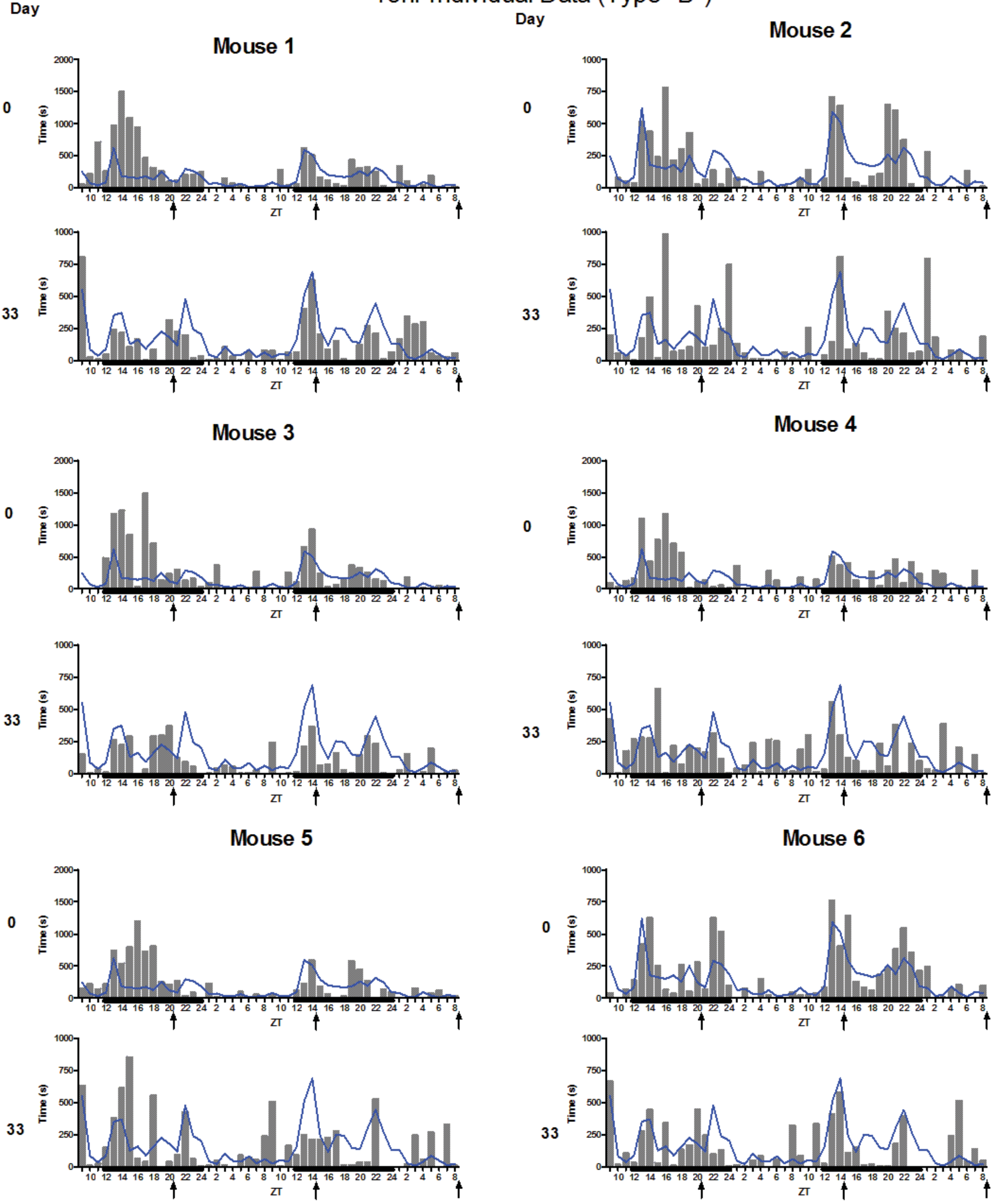

Supplemental Figure 8 (continued)

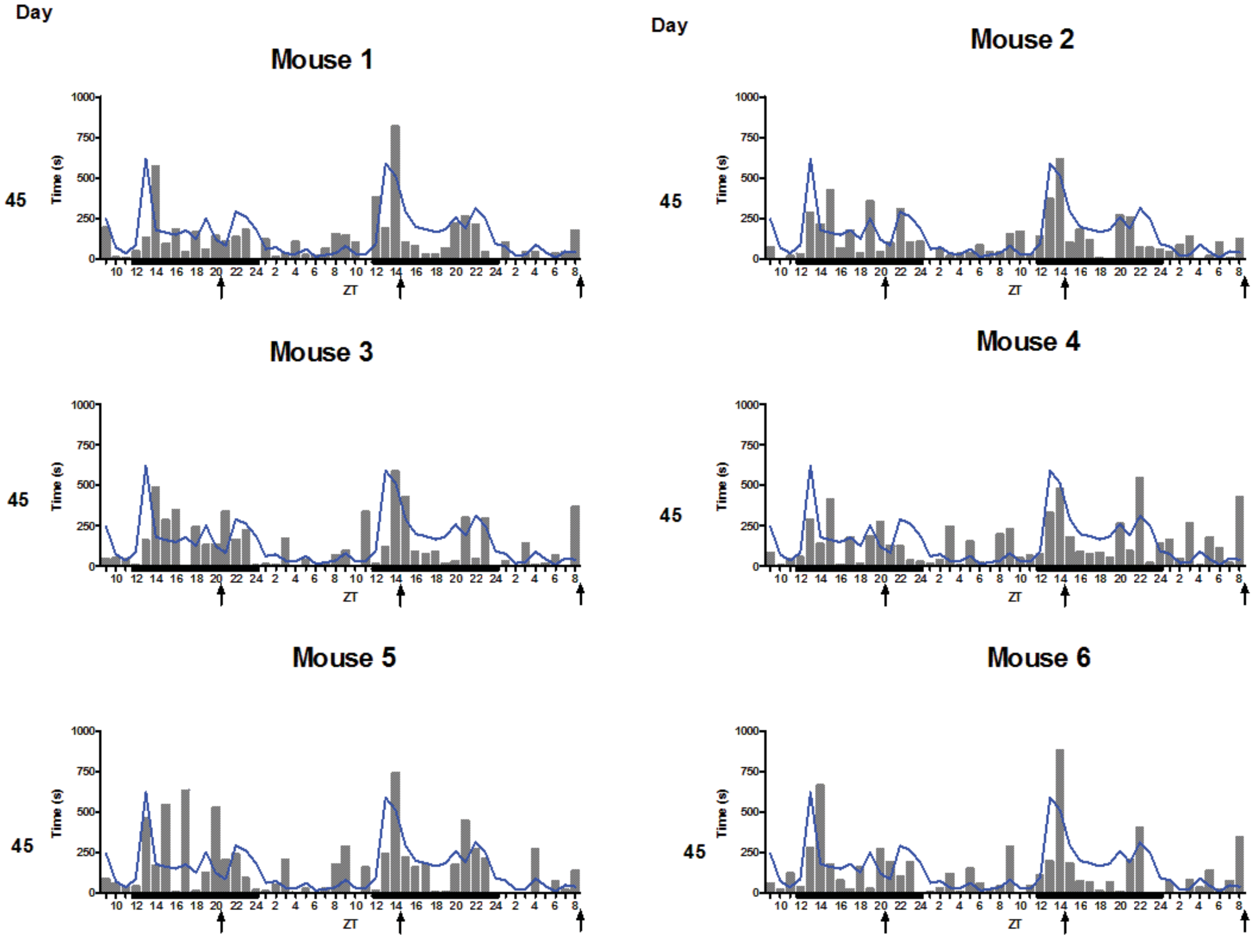

Supplemental Figure 8 (continued)

18 hr Individual Data (Type "C")

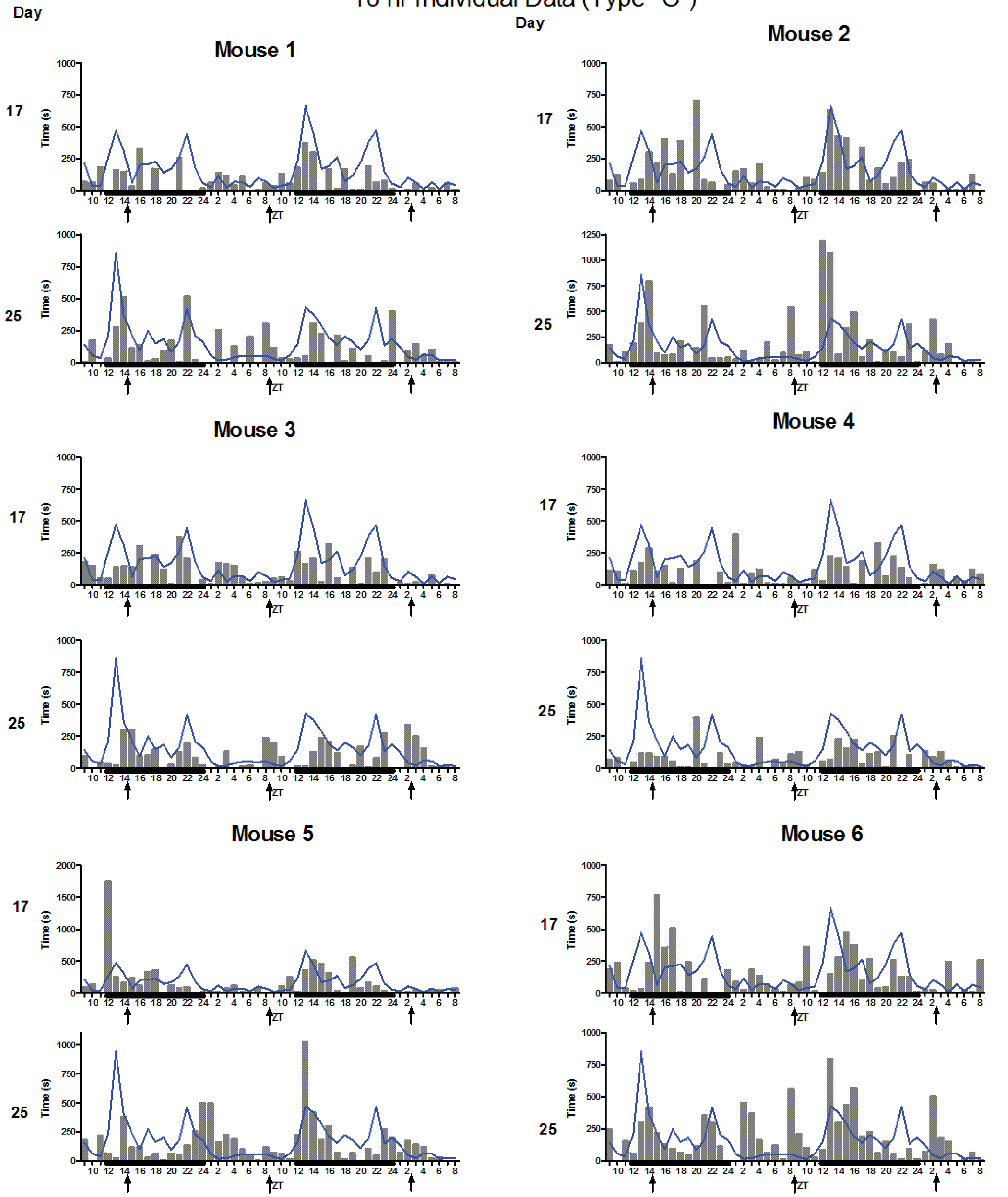

Supplemental Figure 8 (continued)

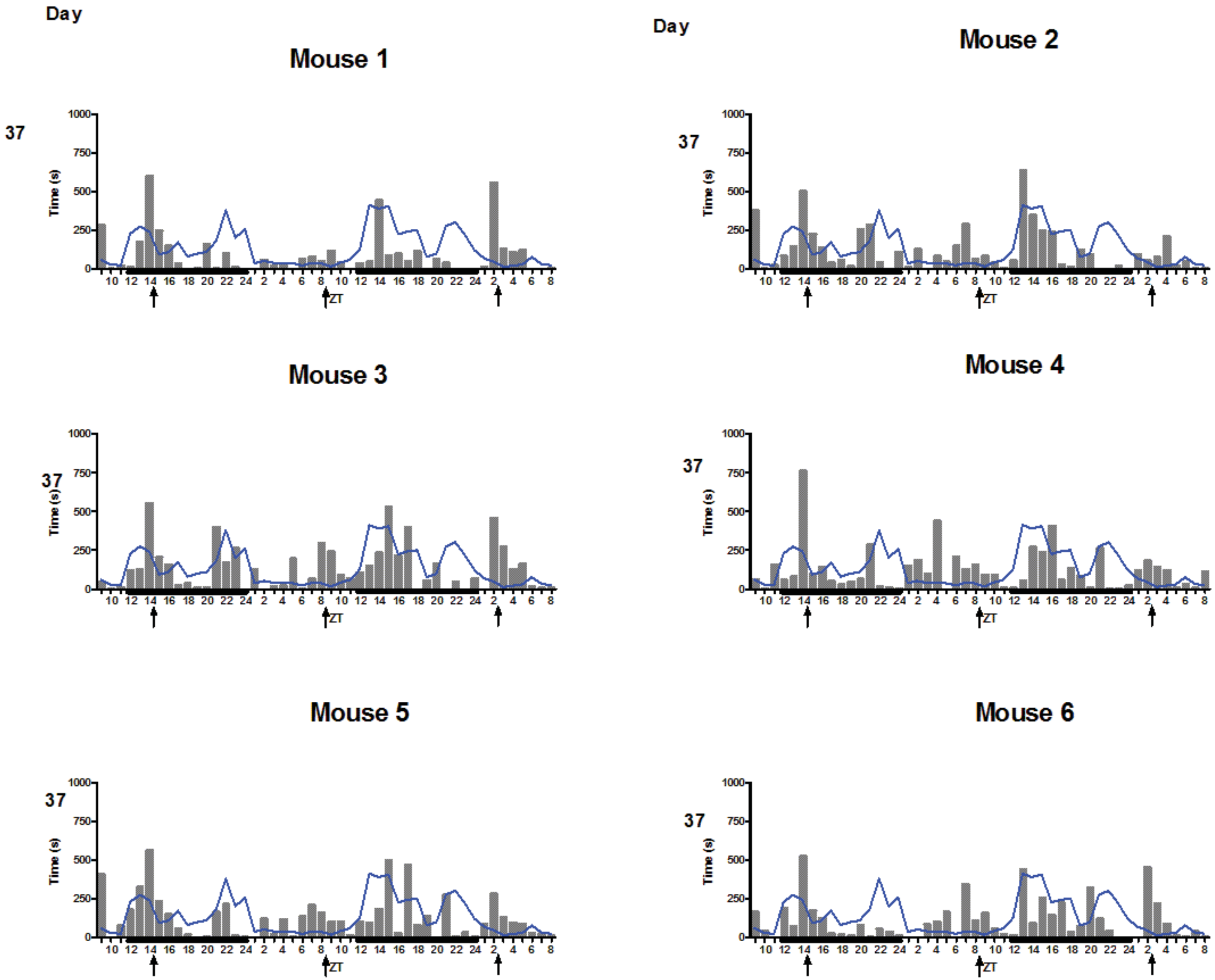

Supplement: Figure S8 — 18 hour interval individual mouse high activity data for days 0–2, 9–11, 17–19, 25–27, 29–31, 33–35, 37–39, 41–43 and 45–47. The feedings are organized by “type”, where “type A” was when feedings occurred at the beginning of a video recording at ZT 9, then at ZT 2, and finally at ZT 20; days 9, 29, and 41 are shown for type A. “Type B” recordings feedings occurred at ZT 20, then ZT 14, and finally at ZT 8; days 0 (the first day of CR), 33, and 45 are shown. For “type C” recordings, feeding occurred at ZT 14, ZT 9, and ZT 2; days 17, 25, and 37 are shown. Anticipatory activity for 18 h interval CR feedings was most visible in type C recordings, possibly because two of the feedings occur during the light cycle. (PDF) [file pone.0037992.s008.pdf]
